# Integrated Medical and Digital Approaches to Enhance Post-Bariatric Surgery Care: A Prototype-Based Evaluation of the NutriMonitCare System in a Controlled Setting

**DOI:** 10.3390/nu17152542

**Published:** 2025-08-02

**Authors:** Ruxandra-Cristina Marin, Marilena Ianculescu, Mihnea Costescu, Veronica Mocanu, Alina-Georgiana Mihăescu, Ion Fulga, Oana-Andreia Coman

**Affiliations:** 1 Department of Pharmacology, Clinical Pharmacology and Pharmacotherapy, Faculty of Medicine, “Carol Davila” University of Medicine and Pharmacy, 050474 Bucharest, Romaniaion.fulga@umfcd.ro (I.F.); oana.coman@umfcd.ro (O.-A.C.); 2 Doctoral School of Biological and Biomedical Sciences, University of Oradea, 410073 Oradea, Romania; 3 National Institute for Research and Development in Informatics, 011455 Bucharest, Romania; alina.mihaescu@ici.ro; 4 Center for Obesity BioBehavioral Experimental Research, Department of Morpho-Functional Sciences II (Pathophysiology), “Grigore T. Popa” University of Medicine and Pharmacy, 700115 Iasi, Romania; veronica.mocanu@umfiasi.ro; 5 Faculty of Automatic Control and Computers, National University of Science and Technology POLITEHNICA, 060042 Bucharest, Romania

**Keywords:** post-bariatric surgery, personalized smart nutrition, digital health monitoring, clinical nutrition management, remote patient-monitoring systems

## Abstract

**Introduction/Objective:** Post-bariatric surgery patients require long-term, coordinated care to address complex nutritional, physiological, and behavioral challenges. Personalized smart nutrition, combining individualized dietary strategies with targeted monitoring, has emerged as a valuable direction for optimizing recovery and long-term outcomes. This article examines how traditional medical protocols can be enhanced by digital solutions in a multidisciplinary framework. **Methods:** The study analyzes current clinical practices, including personalized meal planning, physical rehabilitation, biochemical marker monitoring, and psychological counseling, as applied in post-bariatric care. These established approaches are then analyzed in relation to the NutriMonitCare system, a digital health system developed and tested in a laboratory environment. Used here as an illustrative example, the NutriMonitCare system demonstrates the potential of digital tools to support clinicians through real-time monitoring of dietary intake, activity levels, and physiological parameters. **Results:** Findings emphasize that medical protocols remain the cornerstone of post-surgical management, while digital tools may provide added value by enhancing data availability, supporting individualized decision making, and reinforcing patient adherence. Systems like the NutriMonitCare system could be integrated into interdisciplinary care models to refine nutrition-focused interventions and improve communication across care teams. However, their clinical utility remains theoretical at this stage and requires further validation. **Conclusions:** In conclusion, the integration of digital health tools with conventional post-operative care has the potential to advance personalized smart nutrition. Future research should focus on clinical evaluation, real-world testing, and ethical implementation of such technologies into established medical workflows to ensure both efficacy and patient safety.

## 1. Introduction

Bariatric surgery (BS), or weight-loss surgery, includes procedures that alter the digestive system to reduce food intake or nutrient absorption. These interventions are considered when standard treatments fail and obesity-related conditions become severe. BS not only promotes long-term weight loss but also can improve type 2 diabetes, hypertension, sleep apnea, and other comorbidities. However, success depends on lifelong adherence to dietary changes, physical activity, and follow-up care [1].

Post-operative care is crucial for maintaining weight loss, nutritional status, and overall health. This includes personalized nutrition, lifestyle management, and continuous medical supervision [2]. Patients are at higher risk for micronutrient deficiencies, and adherence to dietary guidance is often low; fewer than half meet protein goals or attend recommended follow-ups, underscoring the need for sustained nutritional support [3].

New developments in technology provide encouraging ways to overcome these constraints. In order to enhance clinical nutrition care, artificial intelligence (AI), machine learning (ML), and Internet of Things (IoT) devices have become more and more popular. The application of AI, ML, and deep learning models to forecast nutritional requirements, personalize dietary treatments, and maximize patient adherence across a range of disease populations, including diabetes and obesity, was illustrated in a comprehensive review. In post-bariatric settings, where dietary needs and metabolic responses are dynamic, these techniques enable adaptive, real-time alterations in care, which is a significant benefit [4].

The usage of AI-based platforms, which were first created for oncology applications like breast cancer diagnosis, has also grown to cover nutritional risk assessment and decision support in complicated care settings, according to a scoping study. These systems show translational promise for surgical recovery and comorbidity management by integrating predictive models to direct personalized food planning and health monitoring [5].

Real-time, multidomain monitoring of food intake, physical activity, and biometric markers is now feasible due to concurrent advances in IoT technologies. By combining sensor data with cloud-based analytics, IoT-enabled feeding systems improved treatment for patients with long-term neurological and cognitive deficits, according to a recent evaluation. These systems offer a paradigm for scalable, continuous surveillance, which is essential for spotting early indicators of behavioral deterioration or nutritional deficits in post-BS patients, even if they were created for a different patient population [6].

To improve care, there is a shift from isolated clinical visits to integrated models. Ongoing monitoring helps manage comorbidities like diabetes and hypertension, enabling timely interventions [7]. The integration of medicine with digital tools enhances care quality [8]. Personalized smart nutrition uses real-time monitoring and adaptive algorithms to tailor diets to patient profiles and behaviors, improving adherence and outcomes [9].

Given the complex post-operative course (nutrient malabsorption, metabolic changes, and behavioral shifts), BS patients benefit greatly from technological support [10]. Traditional follow-ups may miss early signs of complications, while digital tools enable comprehensive, personalized tracking.

A key yet often overlooked component is physical activity. Combined with nutrition, it improves weight maintenance, cardiometabolic outcomes, and mental health post-surgery. Systematic reviews have shown that while post-operative exercise may not significantly affect weight loss within the first six months, substantial benefits emerge after nine months, including greater long-term weight maintenance and reduced risk of comorbidity recurrence [11]. One meta-analysis revealed that patients engaging in ≥150 min of moderate-to-vigorous activity per week achieved on average 4% greater BMI reduction one year after surgery compared to less active individuals [12]. Beyond weight outcomes, regular physical activity enhances cardiopulmonary fitness, helps preserve lean muscle mass (counteracting post-operative sarcopenia), and supports mental health, reducing depression and anxiety while improving quality of life [11]. Despite these advantages, current digital and clinical follow-up tools often insufficiently monitor or integrate physical activity metrics. To fully support recovery in bariatric populations, future systems must incorporate structured activity tracking alongside nutrition and lab monitoring.

Digital tools support this approach. Wearables, mobile apps, telemedicine, and AI enable tailored interventions based on behavior, phenotype, and medical history [13,14]. IoT systems capture data on meals, glucose, and activity, creating a connected ecosystem [15,16].

The relevance of AI and IoT technologies in nutrition care has also expanded into other chronic and high-risk domains. Applications of AI, ML, and deep learning now support dietary decision making and health optimization in populations with cognitive decline, oncology, and cardiovascular risk. For instance, AI-driven predictive models and sensor-integrated platforms have proven useful in the nutritional management of patients with chronic neurological conditions, including Alzheimer’s disease. Similarly, AI-based tools for breast cancer care assist in nutritional risk assessment, energy balance monitoring, and personalized post-treatment recommendations. Including these broader domains reinforces the potential and adaptability of intelligent technologies in nutrition care.

mHealth platforms (apps, wearables, chatbots) improve engagement and allow high-resolution tracking of diet, activity, and biomarkers. Studies confirm that remote follow-up can match in-person care in preventing complications [17,18]. EMA tools reduce recall bias and help detect nutrition issues earlier [19]. Wearables monitor diet, activity, sleep, and vitals, key indicators of recovery. Apps that track diet and exercise foster better adherence and long-term outcomes [20]. Cloud systems integrate this data across devices to guide decisions [15,21].

AI systems analyze large datasets to personalize care. They help with outcome prediction and provide tailored diet recommendations [22,23]. One example is an AI-based app generating smoothie recipes based on health goals and sustainability [24,25,26]. These tools give clinicians real-time insights and support patients in self-management. Digital platforms offer continuous monitoring of nutrition, activity, glucose, and vital signs, enhancing early recovery and long-term care [21]. They also help detect cardiovascular risks via HRV and blood pressure monitoring [27]. Body composition tools like BIA and 3D imaging guide interventions post-surgery [28].

Challenges remain regarding privacy, data accuracy, and limited clinician involvement in tool design. The volume of data is massive, and processing protocols are still evolving. Nonetheless, digital tools hold strong promise in improving post-BS outcomes and long-term care [15,21,29].

Several commercially available digital platforms, like NutriSense, DayTwo, Lumen, and MyFitnessPal, have made significant strides in delivering personalized nutrition through the integration of biometric data. These tools collect and interpret continuous glucose monitoring (CGM) profiles, gut microbiome analyses, and other metabolic markers to offer individualized dietary feedback. Beyond data processing, they serve as motivational and educational platforms, enhancing user engagement in dietary self-management. NutriSense, for instance, pairs CGM feedback with expert dietary coaching, providing users with real-time visualizations of their glucose responses to food and activity. This biofeedback mechanism has been associated with improved dietary compliance and metabolic flexibility, especially in non-diabetic individuals participating in weight loss programs [30]. Similarly, DayTwo leverages gut microbiome profiles to generate personalized dietary advice. Its predictive algorithm has shown clinical efficacy in optimizing glycemic responses and supporting metabolic control in users with prediabetes and metabolic syndrome [31]. Lumen, a portable breath analysis device, estimates real-time substrate oxidation (carbohydrates versus fats) to guide metabolic flexibility coaching. While promising in lifestyle and wellness contexts, its role in structured post-surgical recovery is still being evaluated [32].

On a broader level, digital therapeutics combining CGM feedback with behavioral interventions have demonstrated clinically meaningful improvements in both glycemic regulation and weight control, particularly in type 2 diabetes care pathways [33]. Likewise, IoT-enabled systems integrating multiple sensor data (dietary input, physical activity, and vital signs) are emerging as scalable tools for chronic disease management, offering real-time clinician feedback and facilitating adaptive decision making [34].

Despite their individual strengths, these tools often fall short in addressing the multifaceted and clinically intensive requirements of post-bariatric care. Most focus on general wellness, metabolic tracking, or behavioral coaching but lack systematic monitoring of micronutrient status, structured clinician oversight, or compatibility with multidisciplinary workflows. For instance, while NutriSense excels in CGM-based feedback and dietary coaching, it is not typically integrated into formal clinical pathways or used for micronutrient surveillance. DayTwo’s strength lies in microbiome-informed nutrition, yet its scope often excludes body composition monitoring or dynamic tracking of post-surgical nutritional phases. Lumen provides practical metabolic insights but does not support clinical documentation or long-term recovery protocols tailored to bariatric surgery.

In contrast, the NutriMonitCare system was specifically designed to bridge these gaps by integrating the most effective features from these tools—such as real-time biomarker feedback, personalized dietary recommendations, and user-friendly interfaces—into a unified, clinician-supervised platform. It synthesizes patient-reported symptoms, biochemical values, physical activity metrics, and nutritional adherence into a centralized dashboard accessible to nutritionists, endocrinologists, and general practitioners. This architecture allows the NutriMonitCare system to support interdisciplinary care coordination and apply adaptive strategies based on evolving patient data, providing both clinical depth and scalability. In doing so, it moves beyond the limitations of commercial wellness applications by operationalizing structured post-operative nutrition protocols, with an emphasis on micronutrient management, physical rehabilitation, and behavioral engagement.

Through this design, the NutriMonitCare system illustrates how purpose-built digital infrastructures can not only complement but enhance traditional care models. It builds upon proven technological elements while aligning them with clinical guidelines to meet the complex demands of long-term metabolic recovery following bariatric surgery.

The NutriMonitCare system aims to integrate the most effective elements of these platforms, i.e., real-time biofeedback and tailored dietary insights, within a structured, clinician-led environment specifically designed for the complex needs of post-bariatric patients. It is a digital health system developed to address key limitations in post-bariatric care by offering continuous, patient-centered monitoring. Specifically designed for bariatric patients, the system integrates wearable and non-wearable sensors with cloud-based analytics to monitor parameters such as dietary intake, body composition, cardiovascular markers, metabolic signals, sleep quality, physical activity, and lifestyle behavior. Structured interfaces support self-reporting (diet logs, symptom tracking, and lifestyle journaling), ensuring engagement and complementing objective data. Unlike tools focused on isolated metrics, the NutriMonitCare system synthesizes multidomain inputs into a unified, patient-specific interface for both patients and clinicians. It is currently under laboratory testing, with clinical application planned for the next phase.

This paper presents NutriMonitCare as a prototype digital solution tailored to the complex needs of post-bariatric patients. It details the conceptual framework of the system, monitoring domains, and its applications in personalized nutrition, remote clinical supervision, and interdisciplinary collaboration. While still in development, the system illustrates how digital infrastructures can enhance conventional care and support sustained recovery.

The structure of this article is as follows: Section 2 explains the methodological framework behind the NutriMonitCare system, including clinical rationale, digital architecture, and data flow. Section 3 summarizes integration results across key domains: nutritional, physiological, biochemical, and behavioral. Section 4 discusses clinical relevance, patient engagement, usability, current limitations, and future directions. Section 5 concludes with the core contributions of the system to digitally assisted post-bariatric care.

## 2. Materials and Methods

The methodological approach used in the design of the NutriMonitCare system’s development and any prior assessments are presented in this section. Since there are presently no real patients in the laboratory-based testing stage of the system, our scientific procedure was thoroughly integrated with appropriate international reporting regulations that the EQUATOR Network endorses. In particular, the Template for Intervention Description and Replication (TIDieR) checklist [35] was used to directly layout the intervention components, and the Standards for Reporting Qualitative Research (SRQR) framework [36] was used to tackle methodological accessibility, qualitative accuracy, and procedural understanding.

In accordance with TIDieR guidelines, we gave the intervention a distinct name, the NutriMonitCare system, and in Section 2.1, “Clinical Management Protocols,” we include a thorough justification, concept, and specific objectives. Section 2.2 and Section 2.2.1 provide a detailed description of the materials and procedures used, including the digital monitoring intervention, the Withings devices chosen, the measurement of certain physiological and behavioral parameters, and related monitoring procedures. The providers of interventions are well defined. Specified distribution manners are described in terms of digital dashboards for both healthcare providers and patients (Section 2.3.2). We explicitly outline the framework for dynamically personalizing interventions according to simulated patient outcomes, the frequency and length of interactions, the intervention environment (a controlled laboratory setting with simulated scenarios), and fidelity monitoring across tailored computer-generated alerts (Section 2.3.2 and Section 2.3.3).

The Introduction section and Section 2.1 clearly state the research problem, study setup, research question, and particular study objectives, in accordance with the SRQR framework. The basis and documentation of our approach and supporting reasoning are clear and organized across Section 2. Section 2.2 and Section 2.3 include in-depth information on data collection techniques, sources, procedures, and biochemical parameters of interest. The lack of actual patients means that direct contact between target final users and researchers from the development team cannot be described in depth; nevertheless, this drawback is tackled specifically by stating that communications were simulated using meticulously planned, lab-based scenarios. Data compilation and evaluation methods, such as correlational analyses, interactive dashboards, and health data integration, are clearly documented to guarantee reliability and clarity. Section 2.5, “Ethical Approval”, delves into ethical issues and rigorous data security procedures.

### 2.1. Clinical Management Protocols

In order to maximize weight loss, prevent nutritional deficiencies, and ensure thorough recovery, post-BS patients must receive effective therapeutic therapy. A comprehensive clinical protocol consists of ongoing medical practices for nutritional needs, dietary management, and patient health monitoring. Personalized dietary plans, regular checkups, and long-term initiatives to enhance patient health are frequently included in these regimens. In order to optimize patient outcomes, post-bariatric surgery care recommendations in the US and Europe both place a strong emphasis on a multidisciplinary approach that includes frequent medical examinations, nutritional management, and psychological support. Numerous important studies and recommendations provide comprehensive information regarding various procedures, even if precise requirements may vary.

According to the European Association for the Study of Obesity (EASO), dietary treatment, micronutrient supplementation, comorbidity management, and psychological support are all crucial components of post-bariatric surgical care. They suggest that primary care providers gradually take on greater follow-up responsibilities and advocate for interdisciplinary long-term follow-up. The suggestions also stress how important it is to refer patients with challenging clinical problems to specialized centers [37].

The goal of enhanced recovery after surgery (ERAS) protocols is to improve post-operative recovery through evidence-based treatment plans. Pre-operative, peri-operative, and post-operative guidelines that stress the importance of a multidisciplinary team are part of the procedures in BS. In an effort to improve patient outcomes through standardized treatment pathways, ERAS guidelines have been expanded to cover a range of surgeries, including bariatric procedures [38].

In the UK, the National Institute for Health and Care Excellence (NICE) stresses that bariatric surgery services must follow-up for at least two years. As part of a shared care approach including primary care and specialty physicians, they stand for annual monitoring. These guidelines aim to detect any problems early on and offer comprehensive post-operative care [39].

In 2021, the American Society for Metabolic and Bariatric Surgery (ASMBS) in the United States released new dietary guidelines that focused on maintaining micronutrient levels following surgery. In order to avoid typical post-operative deficiencies, these guidelines emphasize the significance of regular nutritional monitoring and offer supplement recommendations. By showing that patients who participated in regular follow-up, including nutritional counselling and psychological support, had better weight loss maintenance and are more proactive in managing potential complications, the recommendations also highlight the importance of creating customized treatment plans based on each patient’s unique needs [40].

### 2.2. Integrated Digital Monitoring Strategy for Post-Bariatric Care: The NutriMonitCare System

Rapid developments in the field of wearables and connected health technologies are modifying the monitoring and treatment modalities previously used for patients following surgery. These devices continuously track physiological parameters and offer the ability to convert static clinical snapshots into dynamic, longitudinal health profiles. In the context of post-bariatric surgery, the following parameters are especially relevant.

1. *Body composition metrics* (e.g., fat and muscle mass) to track the quality of weight loss;
2. *Cardiovascular and metabolic indicators* (e.g., heart rate, blood pressure, sleep apnea events) to monitor recovery and health risks;
3. *Activity and lifestyle metrics* (e.g., physical activity levels, steps and distance walked) to assess adherence to lifestyle plans.

Digital monitoring solutions must provide data security and privacy along with usefulness. To ensure patient confidentiality as well as secure access solely for authorized individuals, all transmissions are encrypted, and data is processed and stored in accordance with appropriate healthcare regulations regarding privacy, such as the General Data Protection Regulation (GDPR).

Digital ecosystems are set up to gather, send, and evaluate behavioral and physiological data in almost real time during post-bariatric monitoring. Wearable and ambient sensors gather unprocessed information; connectivity layers (like Bluetooth and Wi-Fi) securely send it to a cloud-based platform; analytics elements process the data in order to detect patterns, deviations, or potential hazards; and interfaces give physicians or patients direct feedback. These systems are generally made up of interconnected parts. This ongoing cycle fosters proactive care and makes it possible to tailor medical care or nutrition strategies early on. Although patients interact through user-friendly interfaces that provide practical knowledge, compliance indicators, and wellness-monitoring tools in line with their treatment plans, clinicians have the ability to access organized dashboards and periodic summary reports to assist with evidence-based decision making

Clinically certified devices like smart scales, blood pressure monitors, sleep analyzers, and smartwatches represent some of the digital tools used in these systems. These are chosen because they have been shown to be accurate, simple to include, and pertinent to post-bariatric recovery parameters such as heart condition, activity levels, body composition, and sleep quality. Every device in the present monitoring configuration can wirelessly synchronize data with central frameworks and complies with international medical device standards.

These concepts were put into practice in the current study by creating NutriMonitCare, an original monitoring system made especially for post-bariatric patients. These certified devices are incorporated by the system into one structure for ongoing data gathering, patient interaction, and clinical feedback. The NutriMonitCare system provides a cohesive monitoring framework that links unbiased sensor data with patient-reported outcomes and clinician-driven recommendations, in contrast to many other digital systems that focus on distinct areas such as glucose monitoring, food logging, or physical activity tracking. The research and development team are now assessing the system in a lab setting while it is at an advanced prototype stage. Future clinical integration will rely upon organized testing.

Prior to introducing the overall system architecture, the subsequent sections provide an organized methodical overview of the NutriMonitCare system by outlining its main functional elements. Since they are the main sources of data, the monitoring modules—which include device-based measurements, clinician-generated documentation, customized suggestions, and patient self-reports—are presented first. The architectural model that follows describes how these inputs are structured, secured, and integrated to facilitate clinical decision making. The real development and implementation rationale of the system is reflected in this pattern, from detailed monitoring methods to the overall digital architecture that makes them cohesive.

#### 2.2.1. Clinical Parameters Monitored by Smart Devices

Continuous, real-time physiological monitoring is essential in post-bariatric care, promoting safe recovery and sustaining a long-term weight loss process. The NutriMonitCare system offers a dynamic and clinically rich profile of every patient, using a suite of smart devices to measure key physiological data. The Withings Body+ scale, ScanWatch 2, BPM Core, and Sleep Analyzer transmit data to the digital platform in real time (Table 1). These devices have been selected due to their complete compatibility with the NutriMonitCare system, clinical validation, and patient-friendliness. Every device can automatically synchronize wirelessly, guaranteeing smooth data transfer and interaction with the analytics and reporting modules of the system. These devices are CE-marked medical devices, according to European MDD/MDR standards [41]

Body weight, BMI, body fat percentage, and muscle mass are *body composition data* gathered using the Withings Body+ smart scale. These metrics are used to evaluate both the qualitative composition of weight loss and its quantitative efficiency. For instance, the ideal post-bariatric trajectory involves a decrease in body fat while maintaining or increasing muscle mass. Insufficient physical activity or protein malnutrition may cause muscle loss, which needs to be addressed promptly so as not to cause metabolic complications.

*Sleep quality metrics* including duration, efficiency, apnea events and sleep score are tracked by the Withings Sleep Analyzer. This population has a high prevalence of sleep problems. Following surgery, a decrease in the apnea–hypopnea index may indicate better airway patency due to weight loss, but persistent episodes may necessitate airway pressure treatment.

The Withings BPM Core and ScanWatch 2 measure *cardiovascular parameters* such as resting heart rate, HRV, and blood pressure. Since hypertension is so common within the obese population, blood pressure is among the most important parameters, and a reduction in these values usually indicates better metabolic performance after surgery. Low HRV may indicate inadequate recovery or overtraining in the early stages of rehabilitation.

*Activity and mobility metrics*, such as step count, distance walked, calories burned, but also physiological parameters like SpO_2_ levels and skin temperature are measured by the Withings ScanWatch 2. These metrics are crucial to monitoring adherence to lifestyle advice and physical rehabilitation efforts. They are particularly useful to indicate early warnings of post-operative problems.

Depending on the usage context, information collected by these devices is discreetly and continually gathered. This might range from daily overviews (e.g., weight or sleep reports) to several times per minute (e.g., heart rate). This improves clinical comprehension by making it possible to generate high-quality longitudinal profiles instead of static time-point data.

These validated smart devices interact to create a logical monitoring framework that allows for accurate, real-time post-bariatric recovery evaluation. The findings establish a strong basis for prompt clinical assessments, tailored interventions, and early identification of issues during the course of rehabilitation.

#### 2.2.2. Post-Consultation Clinical Reports

In the NutriMonitCare system, post-consultation clinical reports serve as a crucial component intended to provide organized, reciprocal contact between patients and healthcare providers. These reports are generated by the attending clinician—typically an endocrinologist, bariatric specialist, or a nutritionist—either during the clinical visit or following in-person or teleconsultations. After having been generated, the reports are uploaded to the NutriMonitCare system, where they are securely stored and made available to the patient and every member of the care team through separate interfaces. Contrary to automated summaries, these reports are clinician-authored. This ensures contextualized medical judgement, based on the doctor’s holistic understanding and evaluation of the patient’s current state.

Each report adheres to a standardized format, ensuring completeness as well as ease in interoperability. The structure loosely follows the Subjective, Objective, Assessment, Plan (SOAP) methodology, designed to align with recognized documentation standards [46,47].

The typical structure includes the following.

1. *Clinical Summary and Evaluation*: A narrative overview of the patient’s current clinical status, including physical findings, adherence to the post-operative plan, progress since the last visit, as well as any complications. This helps to contextualize trends from connected devices and biochemical markers.
2. *Diagnosis:* The clinician updates the patient’s clinical diagnosis, or suspected complications, including staging of comorbid conditions. The diagnosis follows the (International Classification of Diseases (ICD-10) standard, 10th Revision, ensuring semantic interoperability and consistency in clinical communication [48].
3. *Treatment Plan and Medication Adjustments:* Detailed updates on both pharmacologic and non-pharmacologic interventions.
4. *Nutrition and Lifestyle Recommendations:* Tailored dietary advice, portion control strategies, hydration targets, and physical activity goals based on current device-measured behavior, nutrient intake, as well as biochemical markers.
5. *Follow-Up Plan*: Scheduling of the next clinical visit, requests for additional lab work, referrals to other specialists.
6. *Patient Notes*: Subjective information provided by the patient during the consultation, including symptoms, emotional wellbeing, and perceived barriers to adherence.

The ability of the NutriMonitCare system to incorporate subjective and objective data is improved, medical evidence is consistent, and semantic interoperability is supported by this structured format. It also makes it possible to monitor clinical rationale across time, which enhances medical transparency and coherence of care.

#### 2.2.3. Personalized Nutrition Recommendations

Digital health tools aim to adapt clinical recommendations specifically to the monitored patient based on individual needs, the type of surgery they have undergone, and their recovery phase. The NutriMonitCare system offers tailored guidance, provided by clinicians and dieticians, to support healing, prevent nutritional deficiencies, and sustain metabolic outcomes. Dietary prescriptions are not automatically generated by the NutriMonitCare system. Rather, it offers a controlled setting where physicians may customize, revise, and provide patient-specific recommendations based on a mix of medical judgment and system-integrated data.

The system provides an intuitive digital interface for both clinicians and patients, allowing nutritional care plans to be delivered in a patient-specific manner. Through a dedicated dashboard that contains daily dietary objectives, progress-monitoring features, and reminders, patients may access their customized programs. Self-monitoring devices facilitate continuous interactive contact with the care team by enabling users to record intake, symptoms, or deviations. The personalization process integrates clinical assessments, physiological monitoring, and nutritional targets set by healthcare professionals. For instance, macronutrient goals, particularly protein and hydration, are prioritized in the immediate post-operative period to support wound healing and prevent early complications. Over time, the system allows for graduated reintroduction of food textures and expanded dietary variety. Intake metrics and behavioral data are continuously updated by interconnected medical devices or patient recorded inputs.

The aim of the NutriMonitCare system is to provide entirely tailored diets, not provide generic dietary recommendations. Physicians individually generate recommendations by integrating every patient’s biometric information, surgical procedure, current metabolic markers, and behavioral assessments. This guarantees that dietary recommendations are customized based on the patient’s recovery stage, physiological profile, and particular needs (e.g., protein goals during the initial healing phases or adjustments for micronutrient deficiencies). As new data is gathered, healthcare professionals may proactively adjust objectives and interventions thanks to the system interface, which facilitates the ongoing improvement of this feedback. As a result, instead of using a traditional approach, the system takes into account many different patient pathways.

Based on the patient’s recovery, laboratory test results, and monitored behavior, clinicians adapt prescribed nutritional suggestions. The recommendations are presented using clear visual interfaces, so as not to overwhelm patients but offer clarity and a friendly interface, while maintaining clinical relevance. Transparency and traceability are crucial aspects of the system. Each recommendation is linked to a clinician-generated record, ensuring medical accountability. This module guarantees that nutritional guidance is based on evidence and constantly adaptable, in line with changing recovery pathways and particular patient demands, by combining several data sources into a professionally monitored process.

#### 2.2.4. Patient Self-Reporting

Despite the sophistication of IoT-interconnected health ecosystems, patient subjective experience remains invaluable and irreplaceable for personalized care. In this regard, the NutriMonitCare system integrates a structured self-reporting interface for patients to record time-stamped symptoms, psychological states, dietary and medication adherence, and behaviors. Daily or on-demand updates can be generated through the system interface’s assisted instructions and checklists, or they can be generated in free-text format.

These inputs allow for the detection of early deviations in wellbeing, nutritional tolerance, and behavioral adherence that may not be evident through device-based monitoring alone. Table 2 outlines the main domains of self-reported information collected within the system, the tools or formats used, and their clinical utility in supporting personalized and proactive care.

Every self-reported input is immediately time-stamped and added to the patient’s digital records, making it available to the healthcare team for unbiased parameters and contextual evaluation. Currently, the system uses these inputs for clinical review, but they could be further used in learning models, to predict patient outcomes, and to generate automated prompts for a more efficient review.

### 2.3. Integration of the NutriMonitCare System

The NutriMonitCare system was developed as a modular digital solution to support multimodal, remote patient monitoring for post-bariatric care, and it was designed to complement clinical care pathways and integrate diverse data sources into a cohesive system that aims to improve both clinical oversight and patient engagement in the current prototype version.

**Figure 1:**
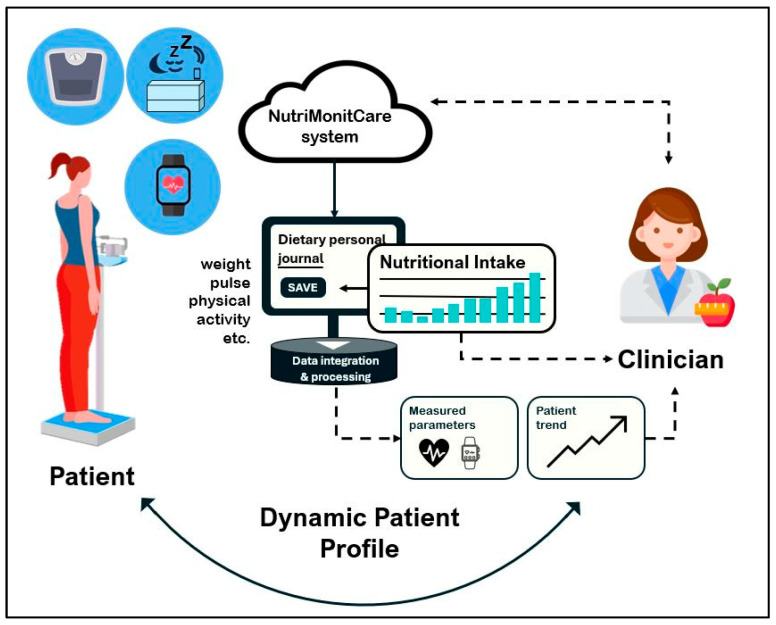
Overview of the NutriMonitCare system. Conceptual illustration of the functional architecture of the system, showing how IoT-based monitoring devices, patient self-reporting modules, and clinician interfaces interact via a central processing and integration platform. The bidirectional data flow highlights the personalized and connected nature of the digital support for post-bariatric nutritional care.

Figure 1 offers a conceptual overview of the system’s functional architecture, providing insight into how NutriMonitCare operates in everyday scenarios. The bidirectional information flow between the patient, self-reporting tools, the primary processing and integration module, IoT-based monitoring devices, and the healthcare professional is highlighted. Prior to going into detail about the fundamental structure and implementation rationale in the subsections thereafter, this conceptual representation illustrates the main system’s components and how they interact.

#### 2.3.1. System Architecture and Data Flow

The NutriMonitCare system includes multimodal data acquisition, from smart devices, patient-reported data, and clinician-entered observations, and currently integrates the following.

1. *IoT environment:* wearable watches, smart scales, and smart mattresses.
2. *Structured patient self-reports*, which are collected through digital forms, patients submit symptoms, appetite, energy levels, bowel habits, mod, hydration and adherence to prescribed nutritional plans.
3. *Biochemical markers* including glucose, lipid profiles, and other lab measurements.
4. *Clinician-generated post-visit reports* summarizing medical evaluations and updates to the care plan.
5. *Clinician-generated personalized nutrition guidance* created by clinicians and logged into the system as a part of individual recovery protocols.

To guarantee consistent data collection, secure transmission, and integrated availability across all monitoring components, the *data flow* of the NutriMonitCare system is divided into four primary stages.

Multiple sources of data are gathered in the initial stage. Wearables and ambient sensors are examples of smart devices that provide physiological data to the user’s mobile application over Bluetooth or Wi-Fi. Simultaneously, patient-reported information on self-assessed symptoms, behavioral patterns, and markers of dietary and treatment adherence is stored using interactive digital forms integrated into the system. Using safe, authenticated interfaces, clinician-generated data inputs are uploaded either manually or semi-automatically. Examples of these inputs include laboratory test results, individualized nutrition programs, and post-consultation summaries.

All gathered data is sent to a centralized cloud-based infrastructure in the second stage. Every data point is encrypted, time-stamped, and associated with a distinct patient profile following receipt. Structured layouts for data storage allow for integration throughout input different types and chronological organization.

Organizing this data into longitudinal patient timelines is the third stage. Clinicians may use this approach to see patterns and changes over time, thereby making it easier to correlate formal medical evaluations, subjective self-reports, and unbiased measurements.

In the final stage, the system provides patients and physicians organized access. In order to facilitate thorough clinical monitoring, healthcare practitioners communicate using a secure, role-restricted dashboard that integrates every useful health parameter and piece of information. During the treatment process, patients may continue storing personal information using an intuitive interface that provides interactive tools, noticeable progress reports, and individualized assistance.

GDPR and other international data protection regulations are complied with fully by all components of the NutriMonitCare system. Although patients have complete visibility regarding their specific medical treatment path and recommendations, role-based access control ensures that only authorized healthcare professionals are allowed to view or alter critical clinical data.

**Figure 2:**
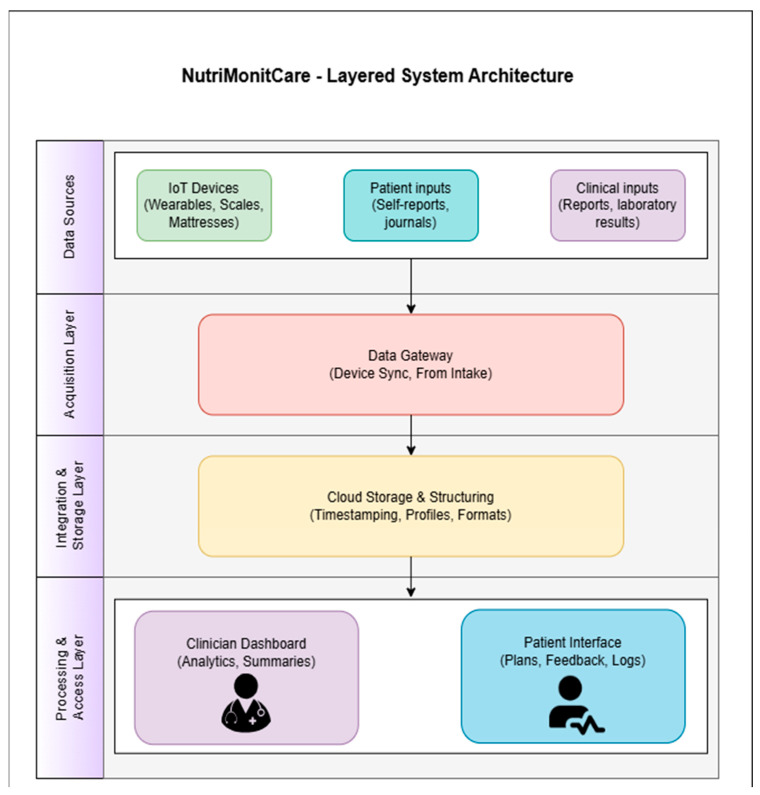
NutriMonitCare’s layered system architecture. Schematic representation of the system’s multilayered architecture of the system, including the data acquisition layer (IoT devices and self-reported inputs), data processing and integration modules, and the interface components for clinicians and patients. The structure illustrates how modularity, interoperability, and compliance with healthcare data standards (e.g., GDPR) are supported throughout the design of the system.

Figure 2 illustrates how NutriMonitCare’s general architecture is organized in a modular, service-oriented model.

The general architecture of the system is further outlined hereafter to support the functional overview of the data flow. The four distinct layers that jointly make up the *NutriMonitCare architecture* are individually in the position of carrying out certain tasks related to the secure collection, processing, and display of behavioral and clinical data that are pertinent to post-bariatric patient care.

IoT-enabled devices, patient-generated content, and clinician-provided content are the three input categories that make up the *Data Sources Layer*. The main clinical, physiological, and behavioral data that compose the cornerstone of patient monitoring are provided by these heterogeneous sources.

The entryway point of the system is the *Acquisition Layer*. The data gateway module, which is part of this layer, is in charge of gathering and validating newly received information from multiple sources. While patient and clinician input are submitted via secure web or mobile interfaces, device-collected data are automatically transferred via Bluetooth or Wi-Fi. Every piece of data that enters this layer is structured and set up for a secure transfer to the subsequent stage.

After being gathered, the data passes on to the *Integration and Storage Layer*, which is made up of a cloud-based, consolidated infrastructure. Here, every piece of incoming data is time-stamped, encrypted, and linked to a distinct patient ID. The system facilitates longitudinal tracking and allows for meaningful association among physiological measurements, biochemical findings, and subjective input by organizing data chronologically and across various forms.

Tailored to roles dashboards are displayed by the system at the *Processing and Access Layer*, which is the last layer. Physicians have access to a secure medical interface that provides summarized patient progress perspectives, including historical data and trend analysis. Patients engage via a distinct capability which provides daily objectives, visual progress reports, and tailored recommendations. The purpose of these interfaces is to facilitate two-way communication and enhance compliance with rehabilitation programs following surgery.

**Figure 3:**
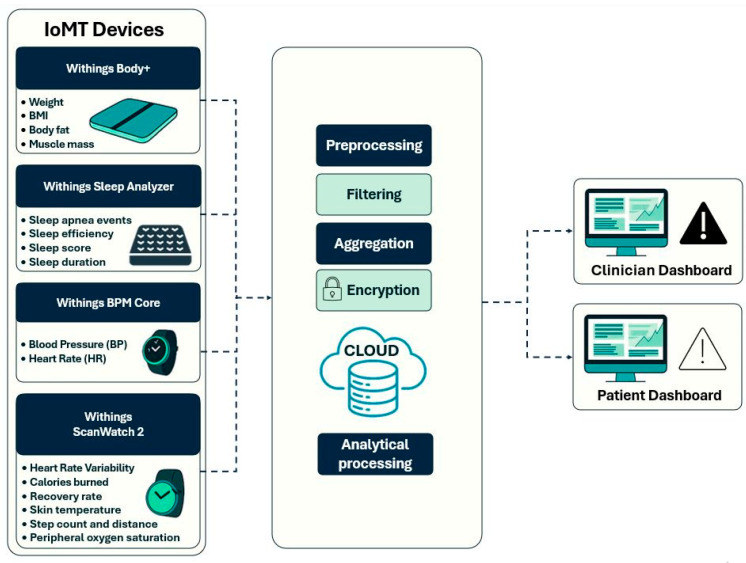
Connected monitoring devices integrated into the NutriMonitCare system. Visual representation of the certified smart devices used for continuous physiological and behavioral tracking in post-bariatric care. The figure shows how multiple data streams—including weight, body composition, sleep quality, physical activity, and cardiovascular indicators—are securely collected via Withings Body+, Sleep Analyzer, ScanWatch 2, and BPM Core. These data are transmitted to the NutriMonitCare cloud platform to support personalized recovery pathways and medical oversight.

The certified IoT-based devices that have been embedded into the NutriMonitCare system are illustrated in Figure 3 to give an even more specific display of how patient data is gathered inside the system. The main data sources are these smart devices, which are used to monitor physical activity, weight, body composition, sleep quality, and cardiovascular health. They allow real-time monitoring to facilitate expert guidance monitoring and responsive medical treatments by gathering ongoing, clinically relevant information. This illustrative overview accomplishes the system architecture by showing how patient-level behavioral and physiological data are collected.

The concept of traceability modularity and compliance with healthcare data security regulations like GDPR is provided by this multilayered architecture. As validation in clinical settings develops, it additionally enables additional capabilities through the inclusion of analytical modules or decision support features.

The NutriMonitCare system was developed with maintainability, scalability and data security in mind, with an emphasis on seamless integration within clinical settings. The backend application was implemented using the Django web framework. This high-level Python 3.12-based platform was selected for its robust built-in tools for user authentication, role-based access control, structured data modeling, and secure request handling. Django’s modular architecture allows for a clear separation of logic, enabling individual components such as patient interfaces, clinician dashboards, and data ingestion modules to be independently developed and managed.

Data persistence is handled using the MariaDB relational database management system. MariaDB supports the structured storage of all inputs, including patient profiles, biometric logs, self-reported symptoms, and clinician-generated recommendations. Django’s Object Relational Mapping layer facilitates secure and consistent interaction with the MariaDB database through Python-based models, rather than direct SQL queries. This approach simplifies data validation and reduces the risk of injection attacks or structural inconsistencies across the system.

The entire platform is deployed on ICIPRO, a national cloud infrastructure providing secure infrastructure as a service and resource. The NutriMonitCare system runs on a virtual machine hosted within ICIPRO cloud’s high-availability environment, which includes redundant networking, load balancing, and real-time failover capabilities. The hosting infrastructure ensures compliance with GDPR through encrypted data transfer protocols as well as strict role-based control over access. This secure cloud environment supports scalable system deployment and accommodates increased user volume as clinical validation progresses.

In a controlled laboratory environment, the NutriMonitCare system is presently going through iterative assessment. As development moves forward, each subsystem—data gathering, synchronization, the clinician dashboard, and the patient interface—is validated separately as part of modular validation processes. Members of the development team conduct these tests, simulating end-user roles (such as patient, nutritionist, or physician) to evaluate system responsiveness, data flow accuracy, and interface rationale. This type of in-lab testing offers a fundamental evaluation of technological resilience and functional consistency, even though it does not yet incorporate actual patient data or clinical deployment. After the prototype is finished, a systematic clinical validation phase involving patients and medical professionals is scheduled.

No real patients have been signed up for use of the NutriMonitCare system at this time because it is still in the laboratory-based prototype stage. As a result, neither clinical sample nor patient feedback are currently available. In order to assess functionality, data flow integrity, and interface design, development team members have performed usability testing by assuming the roles of end users, such as patients, dietitians, and clinicians. The outcomes of these scenarios are not to be considered as representative of genuine effectiveness or medical outcomes, nor do they qualify to clinical trials. Future clinical research will involve comprehensive patient evaluation and real-world validation.

To complement the architectural overview and data flow description, illustrative screenshots of the user-facing and clinician-facing interfaces are included in Section 3. These visual examples highlight the key functional components of the NutriMonitCare system as implemented in the laboratory prototype.

#### 2.3.2. Monitoring and Clinical Integration

This section illustrates how the gathered data flows are clinically carried out using monitoring evidence and proactive outputs, building on the architectural framework previously mentioned. All incoming data streams are securely transmitted to an encrypted cloud environment where they are integrated and then displayed to user dashboards. The system supports the following:

1. *Cross-variable data correlation* for real-time comparison between trends found in physical activity, dietary habits and biochemical markers;
2. *Customizable alert thresholds* for patient personalization, triggered when patient metrics deviate from patient-specific recovery targets;
3. *Visualization of physiological and behavioral metric trends*, enabling clinicians to determine long-term adherence patterns and recovery.

With the use of these characteristics, physicians can shift from occasional encounters to ongoing, data-driven monitoring, making real-time modifications to treatment and dietary recommendations in response to each patient’s particular progress. Relying on the post-operative stage and patient risk level, clinicians usually consider dashboards at predetermined intervals, which might range from weekly to monthly.

Clinicians individually determine the alert degrees at the starting point and dynamically modify them when the patient’s condition changes. Clinical assessment is prompted by triggered alerts and can consist of direct patient communication, nutrition plan adjustments, or laboratory test recommendations. The system is a proficient decision support tool that improves reliability and reactivity in multidisciplinary medical care, even though it does not presently offer autonomous clinical decision making.

Strictly mapping all monitored data to standardized medical terminology (e.g., SNOMED CT, LOINC) allows for possible interface with electronic health record systems (EHR). In accordance with GDPR and relevant healthcare regulations, role-based access control, secure cloud storage, and encrypted communication are used to ensure data protection and privacy throughout the process.

#### 2.3.3. Biochemical Data Integration

Biochemical monitoring is among the most important methods of evaluating the patient’s internal metabolic status during post-bariatric recovery. The NutriMonitCare system integrates periodic laboratory data uploads that are examined in conjunction with device data. These data are manually or semi-automatically uploaded from laboratory results, usually during routine follow-up appointments, rather than being gathered in real time. Given the significantly high risks of nutrient deficiencies, hormonal imbalances, and metabolic changes following BS, this integration renders it possible to link these significant insights with ongoing physiological data from smart devices.

To ensure that clinical interpretation is both comprehensive and actionable, key biochemical markers are grouped into functional categories reflecting their role in post-bariatric recovery. These include indicators of glycemic control, cardiovascular health, micronutrient status, and systemic inflammation. Table 3 summarizes the primary laboratory parameters integrated within the NutriMonitCare system, along with their clinical purpose and how they are contextualized alongside wearable device outputs and patient-reported symptoms.

The data architecture of the system encompasses these lab assessments, which are then compared to patient-reported results and data generated via the device. This renders it feasible to provide a thorough, multisource clinical interpretation which assists in early identification of nutritional or metabolic abnormalities and individualized decision making.

#### 2.3.4. Clinical Dashboard and Alert Rationale

The backend infrastructure collects and aggregates raw data using private *Application Programming Interfaces* (APIs). Synthesized patient summaries are displayed both to the clinicians, and the patients, ensuring adherence and transparency. Results are compared to personal recovery targets, based on the surgical protocol, as well as earlier clinical evaluations.

Threshold alerts are triggered for deviations such as weight regain or plateau in an unexpected post-operative trajectory; insufficient step count, defined by a 3-day average below the activity threshold; or indicators of poor sleep quality, such waking up frequently or an inadequate number of sleep hours. Unusual fluctuations in heart rate or dips in SpO_2_ also trigger alerts, indicating excessive effort or undiagnosed sleep apnea. Similarly, discrete self-monitoring or absence of data might reflect disengaging behaviour or digital exhaustion—a form of user fatigue associated with sustained digital interaction and frequent notifications—which may reduce adherence and long-term engagement [50,51]. This enables streamlined, automated interventions that allow healthcare professionals to observe signs of deterioration in real time.

Clinicians establish every alert criterion at the initial setup process, and they may be tailored according to the patient’s comorbidities, recovery stage, and surgical approach. According to the long-term patterns identified on the patient dashboard, thresholds can be modified manually and are dynamically configurable. Color-coded status indicators, time-series charts of physiological and behavioural parameters, and historical availability of consultation records and prior alerts are just a few of the visualization aspects that make up the clinician-facing dashboard. Customized subsequent follow-up is supported by user-friendly applications that enable filtering by date, variable, or severity. Automated alert updates take place at scheduled times or when new data is uploaded, even if data processing happens almost instantly. Assessment and swift reaction are rendered possible by the prioritization of alerts according to their clinical significance and severity. The alert capability presently utilizes predictable rationale and preset thresholds; after validation, adaptive learning algorithms may be included in later versions.

Semi-automated monitoring procedures are provided by the NutriMonitCare system; nevertheless, every clinical decision remains supervised by qualified professionals. In this respect, “semi-automated” implies solutions that support professionals’ decision making through predefined alerts and pre-established guidelines rather than producing diagnoses or treatments on their own. Such hybrid reasoning improves efficiency while boosting healthcare efficacy [52]. In order to guarantee safe and ethical patient care, the alerting capability is intended to enhance professional medical expertise rather than to replace it.

The architecture of the NutriMonitCare system has been designed for future interoperability with conventional clinical infrastructures, such as EHR systems, even though it is now functioning as a stand-alone prototype in a controlled validation environment. It has the ability to potentially integrate with institutional medical information systems due to its standards-based data structure (such as HL7 FHIR compatibility) and flexible backend. This architecture maintains meaningful consistency for structured data interchange and supports scalable implementation in healthcare processes. After clinical validation, full interoperability—including bidirectional data synchronization and clinical alert transmission to EHR dashboards—is anticipated for further development stages.

### 2.4. Patient Engagement and Data Collection

Long-term compliance with post-bariatric therapy protocols depends on ongoing patient involvement, especially in light of the behavioral, nutritional, and psychological adjustments that must be accomplished. The NutriMonitCare system was set up in order to facilitate active involvement and provide tailored feedback based on structured data gathering.

The ability to facilitate patient engagement and to support tailored post-operative care through structured data collection and feedback is an important aspect of the NutriMonitCare system. To promote sustained engagement, the system provides several key features, detailed as follows.

1. An *Intuitive user interface*, allowing users to easily view and update their own data, including trends in weight, activity, sleep, and biochemical markers. These insights enhance self-monitoring while providing patients with immediate knowledge regarding how they are progressing.
2. *Automated reminders and alerts*: Both configured habits, such daily or weekly input phases, and borderline discrepancies, like extended periods of inactivity or unlogged meals, can cause notifications. To facilitate long-term monitoring of engagement, every user interaction is time-stamped and documented.
3. *Tailored educational content and guidance*, aligned with each post-surgery phase, increase health literacy and adherence to recovery protocols, providing recommendations for hydration targets, dietary improvement, and symptom recognition. Healthcare professionals may adjust this content according to each patient’s level of literacy and rehabilitation plan; it is provided via the patient interface.

The system highlights accomplishments and emphasizes areas that require work with straightforward indications like progress bars and color-coded indicators. These technologies serve as a component of a larger engagement strategy that merges digital feedback with direct medical monitoring, and their purpose is to empower users rather than to replace clinical contacts. The NutriMonitCare system supports proactive patient engagement in the process of recovery and aids in maintaining commitment to long-term health objectives by coordinating behavioral data, automated assistance, and organized clinical guidance.

### 2.5. Ethical Approval

This study is part of an approved PhD research project conducted under the supervision of the University of Oradea. The research protocol was reviewed and approved by the Institutional Research Ethics Subcommittee of the University of Oradea (Approval No. 2/27.02.2025), in accordance with national and international ethical standards for research integrity and responsible innovation, including the Declaration of Helsinki and the European Code of Conduct for Research Integrity [53,54].

As the present study describes the development and initial evaluation of a digital health prototype (NutriMonitCare system), no human participants or animal subjects were involved in the research process. Usability testing and system validation were conducted exclusively through laboratory-based simulations and expert workflow scenarios. Therefore, no procedures requiring informed consent were performed, and no interventions were applied to patients.

All methods and procedures were carried out with full respect for privacy, data security, and institutional oversight.

The NutriMonitCare system has been designed in accordance with ethical principles pertinent to the expected potential application of AI in healthcare, such as transparency, accountability, and direct human oversight, even though the prototype does not presently have any AI features integrated. Appropriate and accurate patient consent will be necessary for gathering data, cross-source integration, and the generation of tailored recommendations as part of the next clinical validation stage.

Privacy potential risks and data overload are acknowledged as ethical issues due to the potential of the system for long-term biometric monitoring. The system includes role-based access controls, data ownership transparency, and patient-facing interfaces that let users examine and comprehend their own data in order to lessen these. Our commitment to sustainable digital health innovation is reflected in these design principles, which are in line with the Declaration of Helsinki, GDPR, and future EU AI regulations.

To maintain scientific accuracy and openness, researchers used laboratory-based scenarios to assess the usability of the system by replicating the interactions between clinicians and patients. To recreate practical use-cases, interactions were closely monitored, and no real patients were engaged.

### 2.6. Reporting Frameworks and Rationale

The layout and contents of this section are in line with globally accepted reporting standards that the EQUATOR Network recommends in order to promote repeatability and guarantee methodological transparency. In particular, we worked in accordance with the following:

- The TIDieR checklist [55], in order to point out the thorough and sequential description of the NutriMonitCare digital intervention, including its components, communication approaches, customizing processes, monitoring fidelity, and argumentation;
- The SRQR [56], in order to facilitate the open sharing of design reasoning, data-gathering methods, researcher engagement with the system, and ethical issues, particularly given the study’s prototype and preclinical stage.

These frameworks were chosen because they can be used for technology-assisted health treatment options that need thorough, standardized reporting but have not yet been used in clinical trials. Their inclusion demonstrates a commitment to the highest standards in digital health research practice and supports the current work’s rigorous scientific approach.

Supplementary File S1 includes a completed TIDieR checklist that supports this research.

## 3. Results

### 3.1. Comprehensive Nutritional Assessments

By putting together patient-reported information, clinician-entered dietary recommendations, and device-measured parameters like body composition, the NutriMonitCare system facilitates thorough nutritional follow-up. In order to boost recovery of tissue and metabolic stability in the early post-operative phase, the system places a strong emphasis on protein intake and hydration tracking. As part of tailored treatment plans, such targets require manual entry into the system by physicians during subsequent evaluations. Weight, BMI, muscle mass, and fat mass are among the body composition metrics that are constantly monitored with the Withings Body+ scale. This enables the medical team to constantly keep watch on the amount and type of weight loss. A notable decrease in muscle mass, for instance, might indicate a lack of protein or physical activity, prompting the doctor to reevaluate the patient’s diet.

Monitoring the evolution of weight loss following BS requires a multidimensional approach, particularly through the assessment of percentage of weight loss, BMI, and body composition. Percentage of S (%EWL) and total weight loss (%TWL) are essential for quantifying the effectiveness of the surgical intervention and tracking trends over time, showing rapid reduction within the first 6–12 months, followed by stabilization or potential regain [57].

BMI serves as a complementary measure to classify obesity-related risk, evaluate progress across thresholds (e.g., from class III obesity to overweight), and monitor for underweight or excessive weight loss (malnutrition risk) [10]. Body composition analysis offers deeper insight into the quality of weight loss, highlighting reductions in fat mass while monitoring for preservation of lean muscle, which is critical for maintaining metabolic health and physical function. The integrated medical and digital monitoring approach allows for early detection of weight loss plateaus, muscle mass depletion, or weight regain, enabling timely clinical interventions and long-term success in post-bariatric care [58].

Digital platforms should be programmed to flag vitamin B12 deficiency (<200 pg/mL), iron deficiency anemia (ferritin < 30 ng/mL or hemoglobin < 11 g/dL), vitamin D3 deficiency (25OHD_3_ < 20 ng/mL), and undernutrition (albumin < 3.5 g/dL) [59]. The integration of nutritional parameter surveillance with weight loss monitoring is essential to prevent micronutrient deficiencies and ensure the long-term safety and efficacy of bariatric surgery outcomes.

The care team leverages the structured interface of the system to promptly update customized nutritional plans. These approaches include accurate dietary options and macronutrient targets tailored to the particular type of surgery and recovery stage of each patient. Through a specific interface, patients acquire their revised objectives and recommendations. They are also asked to record pertinent self-reported information, such as energy levels, satiety, and symptoms that might affect dietary adherence.

The system enables dynamic, clinician-led nutritional management based on reliable metrics and patient input, even if it does not currently provide automated nutrient monitoring. This guarantees that advice continues to be tailored, verifiable, and sensitive to the patient’s changing requirements.

Table 4 summarizes the key nutritional parameters tracked within the system, their sources, clinical significance, and thresholds that trigger clinical intervention or dietary reassessment.

The capability of the system as a clinical support ecosystem is reflected in these monitoring-driven adjustments, which allow for prompt changes to eating habits based on fluctuating physiological and biochemical input.

### 3.2. Integrated Clinical Monitoring and Nutritional Management

In order to enhance the continuity and specific patient tailoring of post-bariatric care, the NutriMonitCare system was designed to complement standard clinical practice with multimodal data aggregation, as well as methods for visualization and feedback. Its architecture offers both patients and clinicians real-time overviews of relevant health parameters, with a particular focus on nutritional trajectories.

The NutriMonitCare system interface is presented in the next set of figures. These screenshots, which were taken from the prototype that was evaluated in the lab, show how patient data, clinical feedback capabilities, and nutritional monitoring components are integrated in real time.

**Figure 4:**
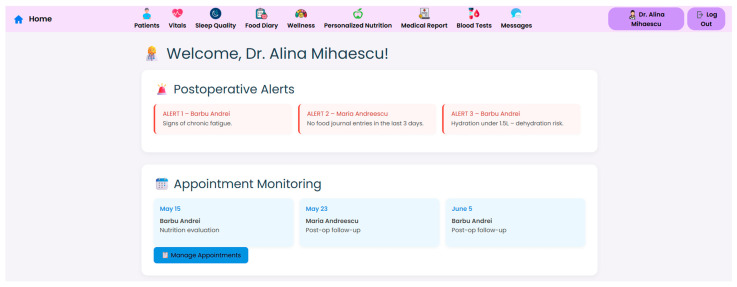
NutriMonitCare clinician dashboard—a visual interface designed for healthcare professionals, integrating key patient parameters such as weight, BMI, biochemical markers, physical activity, and dietary adherence. The dashboard enables real-time monitoring, alert visualization, and coordinated decision making in the post-operative nutritional follow-up process.

At the core of the system is the clinician dashboard (Figure 4), displaying real-time data from wearable devices, patient self-reports, biochemical markers and follow-up reports. The dashboard synthesizes this information into an interpretable interface, supporting care teams in identifying abnormal trajectories and adjusting treatment plans accordingly.

Internationally acknowledged post-operative criteria for bariatric care, including those from ASMBS, NICE, EASO, and ERAS, have been included in the structure and reasoning of the clinician dashboard and patient-facing interface. These frameworks place a strong emphasis on multidisciplinary cooperation, customized dietary counseling, micronutrient monitoring, and longitudinal follow-up. By facilitating frequent evaluations, traceable nutritional plans, and evidence-based goals for recovery and adherence, the NutriMonitCare system converts these recommendations into a useful scenario.

**Figure 5:**
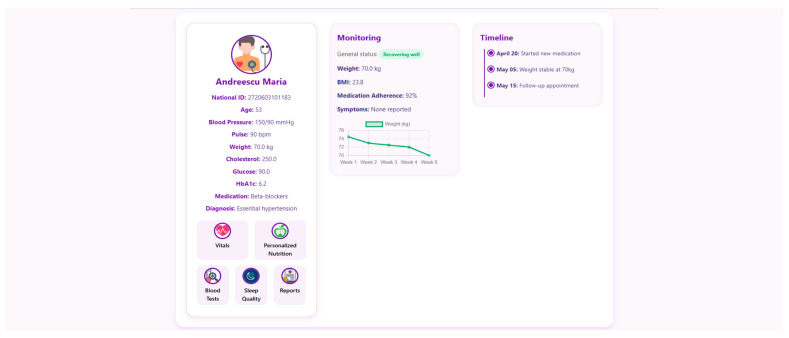
NutriMonitCare patient overview from the “Patient” page—a summary view that allows clinicians to access individual patient profiles, including recent trends in biometric data, risk flags, and follow-up status. This interface supports patient stratification and prioritization within the clinical workflow.

To support efficient clinical decision making, the system offers a structured “Patient Page” that aggregates the most relevant physiological, biochemical and behavioral data for all monitored individuals. This serves as a patient overview, allowing healthcare professionals to assess the most critical post-operative indicators. Each patient entry includes a dynamic timeline (Figure 5), summarizing post-operative events and upcoming consultations.

**Figure 6:**
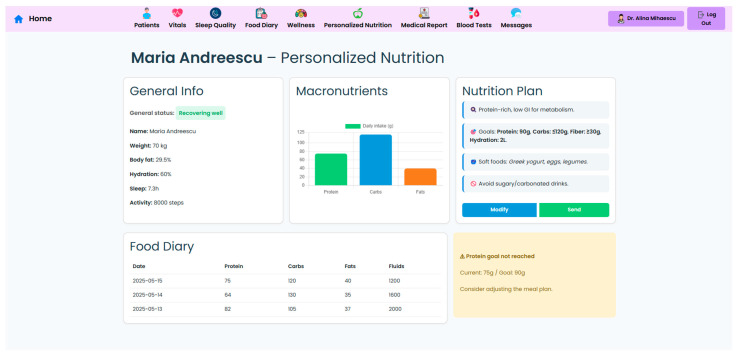
Personalized nutrition clinician module—an interface used by clinicians to view and edit individualized dietary plans based on integrated clinical, biometric, and behavioral data. This module supports nutritional decision making and facilitates tailored interventions aligned with post-bariatric recovery needs.

**Figure 7:**
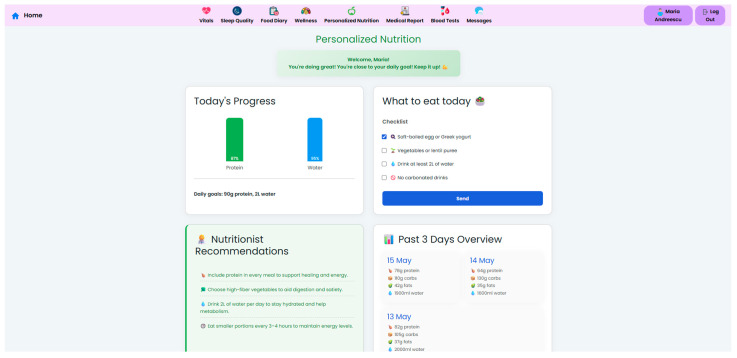
Personalized nutrition on the patient interface—a patient-facing view of the recommended dietary plan, including meal timing, portion suggestions, and hydration goals. The interface promotes adherence by providing clear, structured guidance tailored to the patient’s post-operative nutritional needs.

A key component of the system is the personalized nutrition module, which allows clinicians to send tailored dietary recommendations based on specific patient metrics, surgery type, recent lab results, and patient progression (Figure 6). These nutritional plans are made accessible to patients in their own interface (Figure 7), which also displays the daily targets. Therefore, this bi-directional interface ensures that dietary advice is visible, trackable, individualized, and followed by the patient.

To support adherence, the system incorporates a food diary module, where patients can log their meals and hydration. The system uses these logs, along with wearable device metrics and symptom reports, and generates automated feedback messages that reinforce positive behavior or gently prompt the patient to take correct course actions. Currently, clinician-defined rule-based triggers—such as unfulfilled objectives or identified symptom patterns—are the basis for feedback messages.

**Figure 8:**
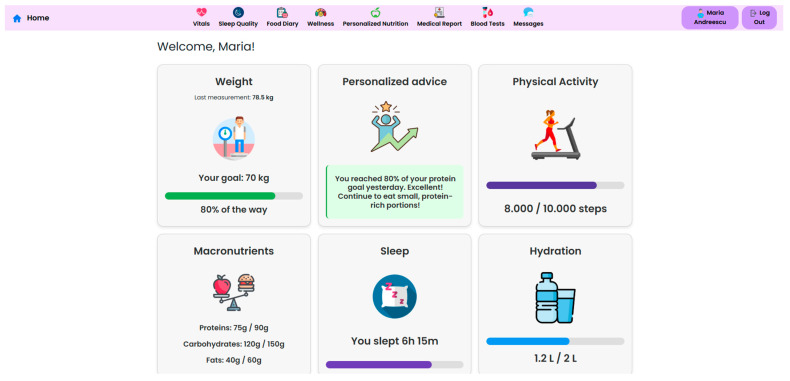
NutriMonitCare patient dashboard—a centralized interface for patients displaying key health indicators such as weight, activity, sleep, and dietary adherence. Designed to enhance self-monitoring, this dashboard supports patient engagement and fosters awareness of progress in the post-operative recovery process.

The NutriMonitCare system offers patients an intuitive dashboard, designed to support engagement and adherence to post-operative care plans (Figure 8). This interface displays personalized messages and nutritional targets along with daily summaries, which are automatically updated using the logged meals. The dashboard also reflects real-time data about physical activity, sleep duration, and adherence to dietary guides. This interactive patient interface ensures continuous self-monitoring through the post-operative recovery phase. While no gamification or behavioral scoring features are currently implemented, the structured visual feedback of the system aims to reinforce adherence and encourage sustained self-monitoring.

By merging self-reported data, device-measured parameters, and clinician inputs into a unified, clinically relevant perspective, the modular dashboard layout aims to lessen data fragmentation. This aids multidisciplinary care teams in providing targeted, customized treatments in the areas of nutrition, behavior, and metabolism. Furthermore, healthcare professionals can accelerate follow-up consultations and improve the efficiency of clinical decision making by using visual indicators and trend graphs to recognize recovery deviations beforehand. Iterative testing by team members acting as end-user representatives was used in preliminary usability evaluations carried out in a lab setting. The assessments concentrated on task efficiency, feedback conciseness, and navigability utilizing scenario-based interactions. The potential of the system for widespread adoption and incorporation into current workflows was further supported by their confirmation that both patients and professionals could easily navigate the interfaces with little to no instruction.

Physicians may download follow-up reports, self-monitoring summaries, and personalized nutrition plans in structured formats (PDF or interoperable medical data standards) using the NutriMonitCare system, which further optimizes recovery processes and fosters consistency of healthcare across healthcare settings. Through integrated communication channels, this feature minimizes data fragmentation and enhances consistent compliance by supporting coordinated follow-up among hospital-based teams and outpatient clinicians.

The NutriMonitCare system was designed for assisting personalized nutritional care in post-bariatric circumstances; it does not automate nutritional decision making. The tool allows doctors to dynamically adapt macronutrient objectives and dietary progression to each patient’s recovery status and operation type by continually gathering physiological data, patient self-reports, and biochemical markers. This feature turns the NutriMonitCare system into a clinical support setting that connects medical expertise with daily monitoring, making sure that nutrition interventions continue to be evidence-based and flexible.

### 3.3. Enhanced Monitoring of Physical and Biochemical Markers

In accordance with the necessities of post-operative care, in addition to nutritional management, the NutriMonitCare system enhances surveillance by integrating continuous, multimodal monitoring of physical activity, biochemical markers, and vital signs. These provide complementary insights into patient recovery trajectories, enabling personalized clinical interventions.

**Figure 9:**
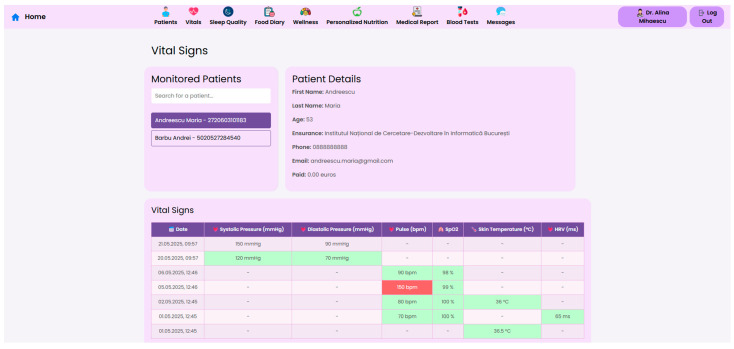

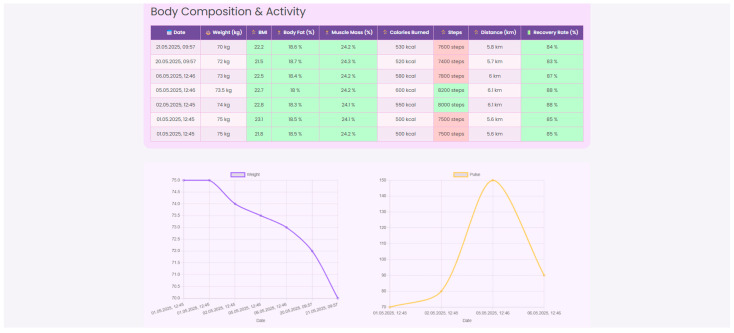
Vital signs page from the clinician’s interface—a detailed view of vital parameters including blood pressure, heart rate, and body temperature, collected via connected devices. This section enables clinicians to track physiological stability and detect deviations that may require timely intervention.

**Figure 10:**
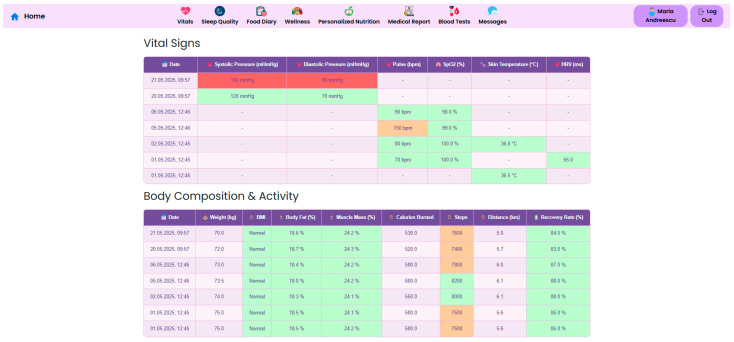
Vital signs page from the patient’s interface—an interface allowing patients to view their own vital sign trends, including blood pressure and heart rate, with simple visual feedback. This page encourages self-awareness and supports active participation in post-operative monitoring.

**Figure 11:**
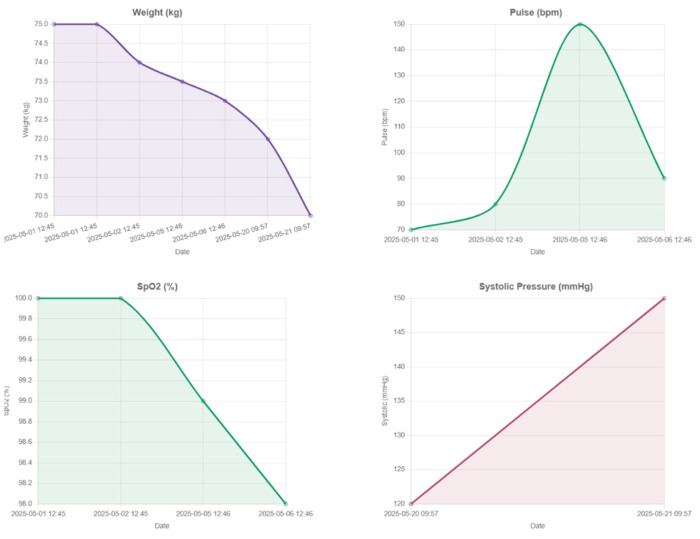
Vital signs trends from the patient’s interface (continued)—an extended view of historical trends in vital signs, enabling patients to observe changes over time. This functionality supports personal health tracking and helps reinforce consistent monitoring behavior in daily life.

Continuous vital sign monitoring is obtained through the use of connected devices to track parameters such as blood pressure, heart rate, SpO_2_, and skin temperature. Beyond traditional vital signs, the system captures dynamic changes in body composition, including weight, BMI, body fat percentage, and muscle mass, essential for assessing post-surgical recovery. Integrated wearable devices record daily energy expenditure, step count, distance walked, and recovery rate, in order to support patient coaching, as illustrated in Figure 9, Figure 10 and Figure 11.

Monitoring the evolution of metabolic parameters such as glycaemia, HbA1c, lipid profile, and liver function is essential for evaluating the physiological impact of weight loss and ensuring long-term post-operative success. In the first 6 months following bariatric surgery, glycaemia and HbA1c should be closely tracked to detect remission of type 2 diabetes, hypoglycemia, or potential glucose fluctuations. In this early post-surgery period, patients often experience rapid improvements in glycemic control, with many achieving partial or complete remission of type 2 diabetes even before significant weight loss occurs [60]. Similarly, improvements in lipid profiles (reductions in LDL cholesterol and triglycerides and increases in HDL) emerge by 6–12 months post-operatively. Long-term follow-up of glycemic markers, particularly fasting glucose and HbA1c, is essential in monitoring the durability of type 2 diabetes remission after bariatric surgery [61]. Following bariatric surgery, nutritional ketosis, often induced by low-carbohydrate or high-protein diets, can be a beneficial metabolic state. However, it is crucial to monitor ketone levels, especially in patients with diabetes or other health conditions, as ketosis can sometimes lead to ketoacidosis. Liver function monitoring, especially ALT and AST levels, is also critical due to the significant reduction in hepatic steatosis that often accompanies weight loss [62]. Regular metabolic assessments, combined with digital weight and activity tracking, can aid in early identification of glycemic deterioration and support timely lifestyle or pharmacological interventions to preserve metabolic benefits.

**Figure 12:**
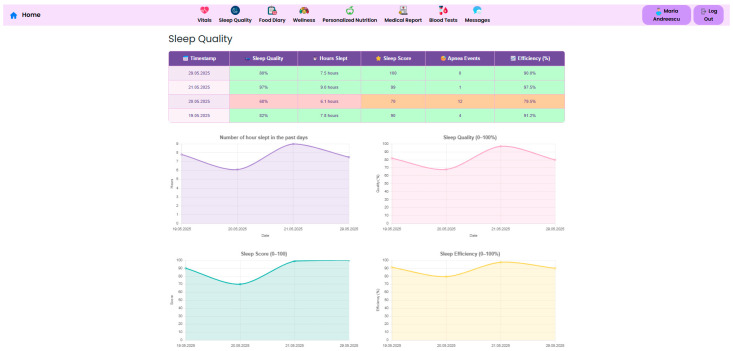
Sleep quality page from the patient’s interface—an interface displaying sleep duration, efficiency, and apnea-related data collected via connected sleep-monitoring devices. This view helps patients to understand their sleep patterns and their relevance in post-operative recovery and overall wellbeing.

Sleep is another crucial component of metabolic and psychological recovery. The NutriMonitCare system includes continuous sleep tracking and offers metrics such as total sleep duration, sleep quality percentage, and apnea events (Figure 12). These metrics are meant to identify abnormalities that may impact hormonal regulation, appetite control and overall wellbeing and may allow early detection of sleep disorders.

Physicians may gain a deeper knowledge of patient behavior and physiology by cross-referencing biometric parameters, physical activity, and sleep quality on a single framework. Low-quality sleep, for instance, may be a sign of after-surgery fatigue or early metabolic dysregulation if it is associated with a lower step count or an increased resting heart rate. Similarly, minor changes in body composition or hydration might inform changes to food and exercise guidelines. A more proactive and detailed plan for recovery strategy, customized to the particular demands and type of surgery of every patient, is made possible by these multiparametric insights.

The system enables the juxtaposition of biochemical data with device-generated parameters, such as HRV or sleep efficiency, in clinical settings where metabolic instability or nutritional deficits are suspected. Instead of depending just on patient-reported adherence or snapshot lab outcomes, this cross-referencing helps clinicians modify macronutrient consumption recommendations or customize supplementation plans according to objective patterns.

### 3.4. Psychological and Lifestyle Adjustments

**Figure 13:**
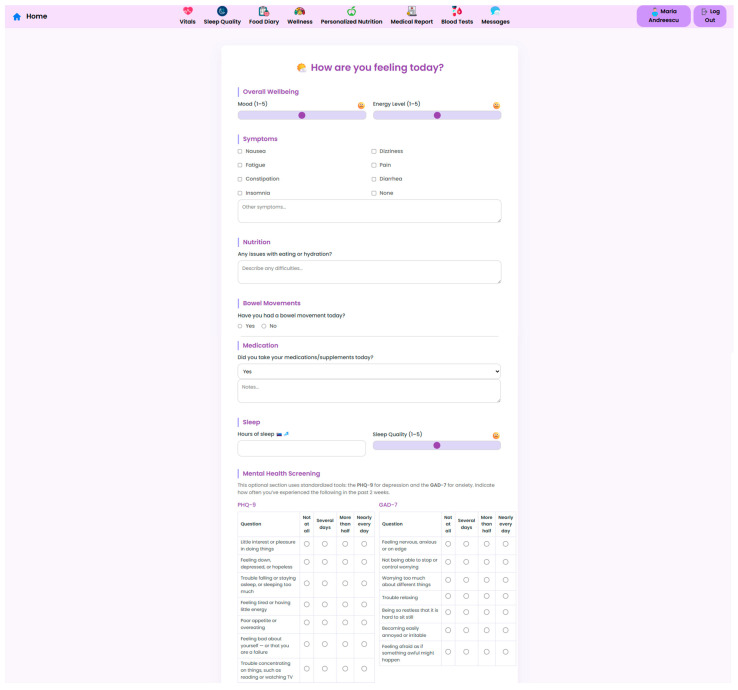
Wellness form for patient self-reports—a structured questionnaire used by patients to report daily wellbeing indicators such as energy levels, mood, appetite, and gastrointestinal symptoms. This form supports the capture of subjective health inputs that complement objective biometric data in personalized care.

The NutriMonitCare system is designed to capture early signs of psychological or lifestyle-related issues that might impact patient recovery. In this regard, the platform includes a comprehensive module for monitoring patient wellbeing. Patients are prompted to report their daily mood, energy levels, sleep quality and presence of common symptoms in the post-operative recovery phase, such as nausea dizziness or fatigue. This input gives clinicians a more nuanced understanding of patient outcomes, allowing them to promptly act when abnormalities are detected. In addition to symptom tracking, tools such as PHQ-9 and GAD-7 are incorporated (Figure 13). These validated mental health screening methods are self-administered and help to identify psychological distress that might otherwise go unnoticed. The integration of these methods supports holistic post-operative care and encourages timely referral to mental health professionals if needed.

The NutriMonitCare system generates an integrated view of the patient’s emotional and physical health through merging behavioral assessment with symptom tracking and physiological monitoring. Through the interface, healthcare professionals can identify trends that can indicate the need for psychological assistance, such as persistently low mood accompanied by poor sleep and appetite loss. Although automated mental health recommendations are not yet available through the system, these understandings aid in quick interdisciplinary responsiveness and knowledgeable assessment.

### 3.5. Interdisciplinary Approaches to Patient Care

**Figure 14:**
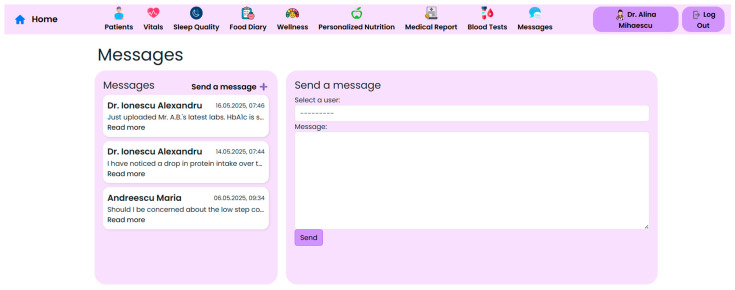
Messaging functionality—a built-in communication tool that allows secure, asynchronous messaging between patients and clinicians. This feature facilitates timely guidance, clarifications, and coordinated follow-up without requiring in-person visits.

Effective post-operative care within the context of bariatric surgery requires close collaboration between surgeons, endocrinologists, nutritionists, psychologists, and general practitioners. To support this collaboration, the NutriMonitCare system integrates a secure interface that allows clinicians to communicate, document, and co-manage patients through centralized access to key records (Figure 14).

**Figure 15:**
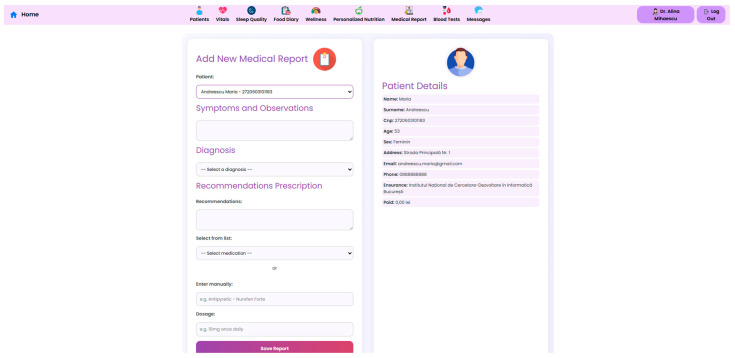
Medical report generation from the clinician’s interface—an interface enabling clinicians to create, edit, and export structured medical reports that summarize patient status, clinical decisions, and recommended actions. This functionality ensures traceability, supports continuity of care, and facilitates interdisciplinary collaboration.

A dedicated functionality of the system facilitates the generation and sharing of structured medical reports, where each entry includes symptoms, diagnostic impressions, treatment recommendations, and prescriptions. Clinicians can either select medications from a standardized database or enter them manually. Each report is linked to a specific patient profile and can be downloaded in a PDF format, simplifying administrative coordination (Figure 15).

The system supports internal communication among providers, offering care teams the possibility to leave notes or flags visible to other members involved in the patient’s recovery. This ensures that changes in medication, psychosocial concerns, or symptoms are visible to all healthcare professionals, promoting continuity of care.

Structured task management components further improve the multidisciplinary capabilities of the system by enabling care teams to assign tasks, monitor outstanding activities (such as lab orders and nutritional adjustments), and receive alerts about patient-specific flags. This lessens the communication lapses frequently seen in post-surgical follow-up and enables a coordinated, real-time workflow. All clinical actions in the system are tracked and recorded, which enhances traceability and ensures that care continuity is upheld throughout the course of recovery.

Development team members have simulated multidisciplinary interactions at the present prototype stage by assuming the roles of clinicians (e.g., endocrinologist, nutritionist, psychologist) or patients. The purpose of these simulations was to assess reasoning for decisions and workflow integration. For instance, an automatic alert for higher HbA1c and less physical activity was sent to an endocrinologist, who adjusted the prescription and marked a request to the nutritionist for macronutrient recalibration. In a different situation, the messaging capability was used to replicate a psychological intervention in response to low GAD-7 mood scores. Dashboard interoperability and cross-role task interoperability have been evaluated using such instances. Real healthcare providers are not involved in the current phase due to some financial and institutional limitations arising from the specificities of the funding program.

Yet, it is anticipated that in subsequent collaborations enabled by other funding sources, physicians will test the completed prototype. Iterative improvements will be based on insights gained from real-world deployment to guarantee alignment with transdisciplinary processes and clinical scenarios.

## 4. Discussion

This study aimed to explore how modern medical techniques and digital health solutions can work together to improve patient outcomes. Healthcare professionals may provide more individualized, effective, and efficient care by combining advanced medical procedures with the newest digital technologies. This synergy enables real-time monitoring of food intake, micronutrient levels, physical activity, and adherence to dietary guidelines, which are key factors in improving outcomes in post-bariatric patients.

The NutriMonitCare system introduces a tailored, integrated framework that adapts dynamically to variations in body composition, self-reported symptoms, biochemical metrics, and physical activity levels. As illustrated in the Results section, clinicians can adapt their recommendations in real time through simulated case workflows that replicate the multidisciplinary decision-making process defined in current bariatric protocols. Comparable digital interventions such as the PromMera app and the BariMEP platform also support self-monitoring and personalized recommendations, though they often lack the deep integration of laboratory and wearable data found in the NutriMonitCare system [63,64]. PromMera is a post-operative mobile health intervention combining physical activity and vitamin/mineral supplementation monitoring. A randomized controlled trial of 154 post-bariatric patients demonstrated increased physical activity and high initial supplementation adherence, although long-term adherence remained unchanged [63]

### 4.1. Medical and Nutritional Strategies in Post-Bariatric Care

Post-operative nutritional care following bariatric surgery requires long-term, individualized interventions implemented by multidisciplinary teams following guidelines issued by ASMBS, NICE, ERAS, and EASO [65,66,67]. The NutriMonitCare system directly operationalizes these frameworks by coordinating real-time nutritional tracking, micronutrient monitoring, physical activity surveillance, and interdisciplinary follow-up in a cohesive digital environment. Recent evidence shows that apps like Baritastic offer robust nutritional tracking and clinic-linked reminders, supporting sustained post-bariatric adherence. A 2024 retrospective study including 4728 sleeve gastrectomy patients reported that higher engagement with Baritastic and being connected to their bariatric clinic were associated with significantly greater percent excess weight loss (EWL) at both 18 months (72.5%) and 24 months (69.3%) post-surgery [68]

In addition to Baritastic, other mobile tools have demonstrated qualitative usability and patient-driven features. For instance, a study found that combining household-tracked data (like weight), wearable activity, and app-based user interactions helped clinicians to better understand patient behavior patterns in post-bariatric contexts [69]. In contrast, the NutriMonitCare system advances these functionalities by integrating laboratory results and wearable-derived data into a unified, dynamic clinical dashboard. This allows it to operationalize structured recovery protocols and facilitate multidisciplinary oversight, capabilities beyond those of current popular apps.

Uploads of laboratory values such as vitamin D, ferritin, and vitamin B12 are not merely stored but actively linked to clinical alerts triggered by concurrent biometric fluctuations or symptom reports. This enables early identification of potential deficiencies or metabolic imbalances, allowing clinicians to intervene before complications arise. Unlike conventional digital tracking tools that focus on reviewing retrospective data, the NutriMonitCare system fosters real-time decision making through structured clinical oversight. A similar approach was seen in the “BELLA” randomized study, where fully remote, app-based follow-up showed comparable results to standard in-person visits in maintaining weight, detecting micronutrient deficiencies, and even achieving higher calcium levels at twelve months post-surgery [17].

While most consumer-facing apps lack this level of laboratory data integration and trigger-based response, the NutriMonitCare system aligns closely with the precision nutrition paradigm, which emphasizes proactive, data-informed clinical management in post-bariatric populations. By evolving dietary recommendations based on ongoing physiological inputs and adhering to ERAS protocols, the system dynamically supports calorie, hydration, and protein needs. This model echoes recent advances in precision nutrition, which leverage biomarker-informed, adaptive dietary strategies to tailor post-bariatric care and mitigate metabolic complications [70].

Moreover, the current ERAS Society guidelines for bariatric surgery advocate for early and individualized peri-operative nutritional intake, particularly sufficient protein and fluids, adjusted in real time according to metabolic and recovery indicators [71].

The interdisciplinary dashboard embedded in the NutriMonitCare system aligns closely with EASO’s recommendation for comprehensive, multidisciplinary care involving dietitians, general practitioners, endocrinologists, and psychologists, strengthening collaboration and reducing the care fragmentation seen in many standalone tools. Recent data from a nine-year Australian cohort show that publicly funded bariatric programs featuring integrated care teams sustainably improve weight and metabolic outcomes, underscoring the value of such collaborative approaches [72].

While the Bariatric Surgery Clinical Decision Tool (BSCDT) similarly advocates for multidisciplinary integration, its scope remains predominantly focused on pre-operative surgical planning and intra-operative decisions, rather than the extended nutritional rehabilitation and long-term follow-up that the NutriMonitCare system supports [73].

Beyond the initial post-operative year, long-term risk remains a critical concern. Nutrient malabsorption, weight regain, and metabolic relapse continue beyond stabilization. The NutriMonitCare system facilitates continuous re-evaluation through structured monitoring cycles, filling a known gap in digital health systems. This mirrors findings from the Baritastic study, which demonstrated that sustained self-monitoring—via regular diet logging, weight tracking, and connection to clinical teams—was associated with significantly greater long-term weight loss and better nutritional oversight after sleeve gastrectomy. Their results highlight the importance of prolonged digital engagement: continuous patient–clinic connectivity correlated with improved adherence and outcomes, reinforcing the value of ongoing of the NutriMonitCare system as a multidimensional monitoring framework [68,74]. The design of the system supports correlation between emerging symptoms and biochemical fluctuations. For example, fatigue paired with dropping ferritin levels may prompt clinicians to preemptively adjust supplementation, reducing the risk of anemia. This is in line with findings from a recent narrative review, which reported that iron deficiency and its associated symptoms like fatigue can persist for years after BS and that structured biochemical monitoring of ferritin is essential for early detection and intervention to mitigate anemia risk [75,76].

The ability of the NutriMonitCare system to differentiate follow-up protocols by surgical procedure represents a key advancement. For patients undergoing sleeve gastrectomy, the emphasis of the system on muscle mass tracking and caloric intake alignment resonates with findings from a 2022 meta-analysis, which documented an average loss of over 8 kg of lean body mass during the first year post-surgery, especially within the initial three months, underscoring the imperative for early muscle-focused monitoring and tailored nutritional interventions like protein supplementation [77].

Conversely, Roux-en-Y gastric bypass (RYGB) patients face distinct risks (vitamin B_12_ deficiency, hypoglycemia, and dumping syndrome) that demand targeted biomarker and symptom surveillance. Indeed, up to a third of RYGB patients experience post-prandial hypoglycemia or dumping episodes, which continuous tracking can identify early and mitigate through dietary or supplementation adjustments [78].

Unlike generic diet or activity apps, the NutriMonitCare system provides this granular, procedure-specific protocol customization, adapting both the nature and frequency of monitoring to match the divergent post-operative challenges of sleeve gastrectomy (SG) versus RYGB, thus significantly reducing the risk of complications through precision follow-up.

The NutriMonitCare system also actively contributes to the prevention of post-operative complications such as sarcopenia, osteoporosis, and cardiovascular dysfunction by embedding alerts for declining body composition, abnormal calcium/vitamin D levels, and irregular postprandial vitals. Unlike apps like Noom or MyFitnessPal, which primarily track diet and exercise without any predictive alert system for clinical biometrics, the NutriMonitCare system offers anticipatory care [79,80]

This approach aligns with clinical evidence; bariatric patients frequently experience rapid declines in lean body mass post-surgery, with one study reporting up to 70% of patients exhibiting sarcopenic indicators within the first six months post-operation, highlighting the critical need for early detection and intervention through regular body composition monitoring [81].

Similarly, “osteosarcopenic adiposity”, the coexistence of muscle and bone loss, has been documented to manifest years after metabolic surgery and is strongly associated with higher fracture risk and cardiometabolic decline, underscoring the value of combining DXA-based bone density checks with muscle mass tracking to enable timely management [82].

In essence, the NutriMonitCare system operationalizes global guidelines by bridging protocol-driven post-operative plans with real-time decision making. It scales into a flexible, clinically integrated platform that extends the long-term success of bariatric surgery far beyond what generic wellness apps can provide.

The modular and interoperable structure of the NutriMonitCare system may be enhanced to other chronic care pathways, such as type 2 diabetes, metabolic syndrome, or cardiovascular rehabilitation programs, where tailored nutritional monitoring is equally vital, albeit this study focused on post-bariatric care.

In clinical practice, the NutriMonitCare system provides a precise answer to the typical problems of maintaining personalized, superior treatment over an extended period of post-bariatric follow-up. It helps physicians by providing them with instantaneous, clinically actionable data, which lessens the need for delayed follow-up visits or patient self-reporting. It improves early clinical intervention and helps stop the progression of nutritional and metabolic issues by turning passive monitoring into an alert-driven system. By combining important biochemical, behavioral, and physiological parameters into a single, easily comprehensible screen, the system lessens the administrative and cognitive strain on providers. This allows decisions to be made more quickly and dietary recommendations and supplements to be customized more precisely. The capacity of the NutriMonitCare system to integrate into existing workflows and support ongoing care beyond the first post-operative year makes it a powerful clinical ally in preventing long-term adverse outcomes such as sarcopenia, bone loss, or micronutrient deficiencies.

### 4.2. Role of Digital Tools in Supporting Medical Practices

Among the most significant current advancements has been the substitution of traditional paper-based diet journals with smartphone applications, offering barcode scanners, food databases, and integration with smart scales and continuous glucose monitors (CGMs). Image-based food logging allows users to take pictures of their meals rather than manually record them, with AI automatically recognizing meals and estimating nutritional values from the photos. Other platforms (like PROTEIN Advisor or UnderMyFork) offer personalized dietary advice or integrate CGM data to visualize glucose responses to meals [15,16,51,83].

The NutriMonitCare system distinctly stands out by being purpose-built for bariatric patients and delivering clinical-grade data integration. Unlike generic wellness applications, it offers seamless capture and interpretation of lab results, body composition metrics, and symptomatology, feeding these into real-time clinical algorithms. A recent systematic assessment of bariatric-focused apps in Germany concluded that most existing tools suffer from moderate-to-low content quality and lack evidence-based sourcing, with no app providing comprehensive medical integration or standardized clinical pathways [84,85].

In contrast, NutriMonitCare embeds validated protocols, contextualizing data for clinical decision support rather than just self-management. Moreover, clinical evidence demonstrates that smartphone-based remote follow-up programs, equipped with structured monitoring schedules, can deliver outcomes equivalent to in-person follow-up after bariatric surgery [17,86]. This positions the NutriMonitCare system not as a mere tracking tool but as a robust digital health intervention that aligns with standard post-operative care. Its design bridges a historically observed gap: the absence of apps that combine long-term metabolic surveillance with immediate, biochemistry-driven clinical insights tailored for bariatric populations.

The NutriMonitCare system combines automated alerts, real-time data tracking, and care team interfaces, fully aligning with post-surgical recovery frameworks and enabling physician engagement in interpreting lab and lifestyle data as it emerges.

Physical activity monitoring forms a cornerstone of this model. Studies consistently show that post-operative exercise improves weight stabilization, body composition, psychological health, and long-term metabolic control. A 2020 longitudinal observational study found that patients who increased their physical activity two years after bariatric surgery achieved significantly greater weight loss, gains in fat-free mass, enhanced cardiorespiratory fitness, and better quality of life compared to those who maintained or reduced activity levels [87,88]. The NutriMonitCare system captures this critical activity data by integrating wearables and ecological momentary assessment (EMA) to monitor step counts, fatigue, and sedentary behavior. Its approach mirrors the BariFit pilot, a mobile health intervention for post-bariatric patients, which demonstrated feasibility and sustained engagement; participants increased daily steps by about 1800 and achieved over 95% wearable adherence across 16 weeks [89].

Notably, BariFit identified links between reduced physical activity and low protein intake and mood disturbances, reinforcing the value of the NutriMonitCare system in enabling early detection and intervention that includes nutrient and behavioral adjustments.

On top of physical activity data, the NutriMonitCare system integrates AI-driven insights and predictive analytics. Similar to platforms like DayTwo and NutriSense, which adapt diets based on glucose and microbiome data, [90,91], the NutriMonitCare system aggregates behavioral logs, symptom reports, biochemical measurements, and activity profiles for comprehensive patient monitoring.

While consumer metabolic analyzers like Lumen [92] offer isolated metabolic feedback, they lack clinical integration or EHR compatibility. In contrast, the NutriMonitCare system centralizes metabolic, lifestyle, and clinical data within a team-based care setting. Addressing known interoperability and data governance barriers, given that only ~11% of health apps are EHR-compatible, it supports role-based dashboards, automated alerts, structured documentation, and future-ready EHR integration pathways.

The ability of the NutriMonitCare system to convert distinct data streams into clinically useful insights helps clinicians by facilitating prompt interventions and collaborative decision making. It is designed for use in organized clinical settings, as opposed to consumer-focused apps, and enables medical professionals to keep an eye on patterns that could otherwise go unnoticed, such as the relationship between mood, physical activity, and dietary intake. This makes it possible to identify adherence problems and concerns regarding relapse earlier. Crucially, its connection with EHR systems improves communication across multidisciplinary teams and improves documentation accuracy and continuity of treatment. From a workflow perspective, NutriMonitCare reduces reliance on disconnected wearables or fragmented patient logs by offering a single platform that facilitates evidence-based, real-time modifications to patient treatment regimens. In crowded clinical settings, where efficient, well-coordinated, and data-driven follow-up may greatly enhance results and sustained engagement, this degree of interoperability is essential.

### 4.3. Patient Engagement and Compliance

A critical determinant of success in post-bariatric care is sustained patient engagement throughout recovery and maintenance phases. Digital solutions such as NutriMonitCare are designed to foster this continuity by enabling bidirectional communication between patients and care teams. Instead of relying solely on retrospective self-reporting during clinic visits, the NutriMonitCare system integrates real-time self-reporting tools, personalized dashboards, and automated reminders to enhance adherence and behavioral accountability.

One of the system’s important strengths is its provision of real-time visual feedback and context-specific educational prompts. These features reduce cognitive load during early behavior changes and reinforce positive habits. This aligns with digital behavior change research, which identifies self-monitoring, goal setting, prompts, and descriptive feedback as key techniques that support habit formation and sustained engagement over time [93].

Moreover, the NutriMonitCare system empowers patients to take an active role in their care by presenting structured data, clear goals, and progress trends. Direct care team interfaces enable collaborative goal-setting and timely interventions, fostering shared responsibility. Patient-centered design research in bariatric populations emphasizes the importance of surgery-specific content, self-monitoring, and accessible trust-based information—factors that resonate with the architecture of the NutriMonitCare system [14].

Nevertheless, while the NutriMonitCare system shows strong potential to enhance adherence, its long-term effectiveness in sustaining behavioral change requires empirical validation. Many digital adherence tools decline in impact over time due to the effects of novelty or user fatigue. Therefore, future longitudinal studies should assess whether engagement with platform features—like automated prompts, educational modules, and progress-tracking—can be upheld beyond the early post-operative period and if such sustained engagement correlates with improved metabolic, nutritional, and psychological outcomes compared to traditional follow-up pathways.

From a clinical perspective, maintaining patient motivation and adherence over time is one of the most enduring problems in post-bariatric treatment, and the NutriMonitCare system provides a workable answer. Instead of depending entirely on patient initiative, the approach allows doctors to reinforce treatment goals throughout crucial recovery periods by integrating engagement tools directly into the care process. Its real-time updates and two-way communication enable clinicians to take early action in response to indications of psychological distress, regression, or disengagement. Stronger therapeutic partnerships are supported by this degree of ongoing, low-friction communication, which also enables more complex patient-specific counselling. Clinically, this can lower dropout rates, increase adherence to physical activity or supplements goals, and improve follow-up consistency, all of which have a direct influence on quality of life and long-term surgical success. In actual use, the NutriMonitCare system improves adherence while also giving clinicians a deeper understanding of behavioral patterns that are sometimes overlooked by conventional follow-up approaches.

### 4.4. Limitations

Despite its robust digital infrastructure, the NutriMonitCare system remains in a prototypical stage and has not yet been tested in real-world clinical scenarios. Current findings are based solely on laboratory testing and structured usability simulations, which—although informative—may not fully reflect the practical complexities of real-world healthcare settings. This significantly limits the generalizability of the findings, and further clinical validation across varied settings is essential.

The lack of in-person patient testing and real health data is a drawback. Although role-based simulations were used for initial usability testing, no actual patients have yet to engage with the system. Consequently, important elements like patient satisfaction, involvement, and perceived utility are not quantified. During the proposed clinical validation phase, which will involve both outcome-based evaluations and systematic feedback collection, these variables will be addressed specifically.

One of the limitations in the system’s earlier iterations was the lack of integrated physical activity monitoring, a critical omission considering the strong evidence supporting exercise as a cornerstone in long-term post-bariatric recovery. While this functionality is currently being integrated into the updated system, the absence of this component in the prototypical version represents a significant gap in multidimensional patient follow-up, particularly as guidelines consistently emphasize physical activity alongside nutrition for optimal outcomes.

In terms of technical and implementation challenges, the system faces hurdles related to device heterogeneity and data interoperability. Different data standards and frequent updates from hardware manufacturers complicate seamless integration. At present, the analytical functionality is largely limited to data visualization and trend observation, despite incorporating diverse input sources such as biochemical results, self-reported patient data, and IoT-derived measurements. Predictive analytics, automated clinical alerts, and adaptive decision support algorithms, which are found in more advanced commercial platforms, are still under development and have not yet been tested within the NutriMonitCare system.

The quality and completeness of data also depend on patient compliance with self-reporting, which may fluctuate due to factors such as digital literacy, access to reliable internet, or user fatigue. Some data, particularly biochemical values, must still be entered manually, requiring patients or clinicians to rely on additional applications, which may introduce errors or omissions.

Unlike AI-driven solutions such as Lumen or NutriSense that offer automated feedback and metabolic insights but lack clinical traceability, the NutriMonitCare system is designed for structured, clinician-led monitoring with high transparency and accountability. This focus on medical coordination and bidirectional data flow offers advantages for long-term integration but also slows down large-scale deployment, especially in institutions that require automated systems for operational efficiency.

Additionally, the system has not yet undergone extensive co-design iterations with diverse patient and clinician groups, which may affect adaptability. Future versions must address practical implementation concerns such as user training, workflow compatibility, and regulatory compliance to ensure successful clinical integration. The reliance of the NutriMonitCare system on patient engagement and consistent input introduces variability in data accuracy and completeness, issues that must be mitigated through improved adherence strategies and real-time feedback mechanisms.

These limitations underscore the need for expanded research to evaluate the platform’s true impact on clinical outcomes, treatment adherence, and integration into existing healthcare infrastructures. Unlike many general wellness apps, the NutriMonitCare system is uniquely tailored for bariatric care, offering structured documentation, clinician supervision, and modular design capable of embedding into traditional and remote care pathways. Its architecture is particularly suited for decentralized follow-up programs, where patient autonomy and asynchronous oversight are essential. The system supports continuous monitoring while reducing the need for frequent in-person visits, aligning with the growing demand for hybrid and telehealth models in post-operative care.

### 4.5. Future Work

The NutriMonitCare system’s future development will concentrate on improving its scalability, usability, and clinical significance. Integrating ML and predictive analytics algorithms that may recognize early indicators of psychological distress, nutritional deficits, or deviations from expected recovery trajectories is one of the top priorities. These features will be created using real-world data gathered from future clinical validation research. Workflow integration will be improved, and manual data entry will be decreased with expansion to include interoperable interfaces with laboratory information systems and EHRs. In order to improve compliance and decrease the rate of abandonment, an additional field of research is improving patient engagement components using adaptive education content, conversational agents, and behavior-based nudging approaches. To assess the performance of the system in enhancing patient acceptance, long-term weight stability, and nutritional outcomes, multicenter clinical studies are envisaged. The NutriMonitCare system will evolve into an efficient, evidence-based tool for digitally enhanced post-bariatric care with the backing of these planned strategies.

The integration of digital health technologies marks an important step toward more individualized, data-driven, and patient-centered healthcare models for post-bariatric treatment. Systems like NutriMonitCare have the ability to close long-standing disparities between clinical procedures, patient behaviour, and long-term outcome monitoring as they shift from controlled settings into real-world practice. Such platforms may improve surgical recovery and rewrite standards of care for patients undergoing metabolic and nutritional transitions by combining technology innovation with clinical knowledge and patient involvement. The full potential of digital health in promoting long-lasting, superior results following BS will require ongoing multidisciplinary interaction, clinical validation, and system improvement.

As part of the forthcoming clinical validation phase, a small-scale pilot study is presently being designed to increase the reliability of the system and its utility in healthcare. In order to assess the user experience and interface efficacy, this will include carrying out formal usability testing with real patients and healthcare professionals using certified tools like the System Usability Scale and Net Promoter Score. Engagement indicators will also be monitored, including the frequency of logins, the completion of self-monitoring activities, and the reaction to feedback prompts. The pilot study will give preliminary insights into perceived benefit, convenience of use, and the potential of the system to increase post-operative adherence when incorporated into genuine clinical workflows, even if comparing results with standard treatment pathways is not yet possible in this phase of development.

## 5. Conclusions

The NutriMonitCare system represents a promising prototype of an integrated digital health system tailored to the specific demands of post-BS care. Designed with real-world clinical utility in mind, it addresses multiple domains of follow-up, including nutritional status, biochemical trends, physical activity, and patient-reported symptoms. By supporting both structured self-monitoring and interdisciplinary coordination, the system supports a scalable model for delivering personalized smart nutrition.

In clinical practice, such platforms could enhance conventional follow-up by reducing reliance on in-person visits, improving early detection of nutritional deficiencies, and supporting timely therapeutic adjustments. Its design is particularly suited for remote and outpatient bariatric settings, where regular monitoring and patient engagement remain challenging. Unlike commercially available wellness tools, the NutriMonitCare system integrates validated clinical indicators, customizable alerts, and bidirectional communication to enable traceable and medically supervised care.

However, as the system remains in a prototypical stage, further work is required to validate its usability, safety, and long-term effectiveness in clinical populations. Real-world testing, patient feedback integration, and ethical oversight will be essential steps in moving from simulation to implementation. Future studies should also assess how digital systems like NutriMonitCare influence adherence, multidisciplinary workflows, and long-term outcomes across diverse bariatric populations.

Ultimately, the convergence of personalized nutrition, digital monitoring, and clinical oversight represents a compelling path forward in the management of bariatric patients. When thoughtfully integrated, such systems can enhance clinical decision making and support patients throughout their recovery and long-term weight management.

## Figures and Tables

**Table 1:** Overview of the digital monitoring devices used in the NutriMonitCare system and their clinical applications in post-bariatric care.

Device	Parameter	Clinical Relevance	Ref.
Withings body+	Weight	Primary metric for measuring bariatric efficacy and tracking weight loss	[42]
	BMI	Standardized metric for assessing the severity of obesity and treatment progression	
	Body fat	Assesses adipose tissue reduction, and metabolic treatment progress	
	Muscle mass	Reflects protein adequacy and guides exercise regimens	
Withings sleep analyzer	Sleep duration	Assesses recovery potential and circadian regulation	[43]
	Sleep score	Reflects sleep quality and continuity	
	Sleep apnea events	Detects obstructive sleep apnea, a comorbidity in obese populations	
	Sleep efficiency	Evaluates sleep quality and potential disruptors	
Withings BPM core	Blood Pressure (BP)	Detects hypertension and cardiovascular risk, which is important in metabolic syndrome	[44]
	Heart Rate (HR)	Reflects exercise tolerance and overtraining	
Withings Scanwatch 2	HRV	Monitors stress, sleep, and behavior changes	[45]
	Calories burned	Essential in guiding dietary adjustments and monitoring adherence to post-op plans	
	Step count and distance	Tracks lifestyle and mobility improvements	
	Peripheral oxygen saturation (SpO_2_)	Tracks oxygen saturation and is relevant in assessing post-op complications	
	Recovery rate	Useful in tailoring post-operative physical activity plans	
	Skin temperature	May reflect a pathological event or stress	

BMI—body mass index; BP—blood pressure; HR—heart rate; HRV—heart rate variability; SpO_2_—peripheral oxygen saturation.

**Table 2:** Dimensions of patient self-reporting in the NutriMonitCare system.

Parameter	Examples	Clinical Use
Symptoms	Nausea, vomiting, fatigue	Detect complications, food intolerance, with emphasis on gastrointestinal features
Mental health	Patient health questionnaire-9 (PHQ-9); generalized anxiety disorder-7 (GAD-7)	Screen for anxiety, depression since there is a high prevalence of mood disorders among post-bariatric surgery patients [49]
Satiety and energy	Daily logs	Detect inadequate intake or dumping syndrome
Medication adherence	Self-log, cross-checked with laboratory results	Evaluate real-life compliance
Journaling	Free text reflections	Monitor and record emotional recovery and adherence motivation; encourage long-term adherence and assessment of psychological state

**Table 3:** Key biochemical markers monitored in the NutriMonitCare system and their clinical applications.

Biomarker Category	Monitored Parameters	Clinical Purpose	Interpretation/Contextual Integration
Glycemic control	HbA1c, fasting glucose, postprandial glucose	To evaluate type 3 diabetes status (remission or recurrence) and detect post-surgery hypoglycemia/dumping syndrome	Especially relevant in Roux-en-Y gastric bypass
Lipid profile	Total cholesterol, LDL, HDL, triglycerides	To assess cardio-metabolic improvement following weight loss	Interpreted alongside blood pressure and cardiovascular vitals from BPM Core and ScanWatch 2
Micronutrients	Iron, calcium, vitamin D, vitamin B12, zinc, magnesium, folate	To detect nutrient deficiencies due to malabsorption or reduced intake	Fatigue, muscle weakness, or ↑ HRV may signal deficiency; prompts targeted biochemical re-evaluation
Liver and inflammatory markers	CRP, ALT, AST, GGT	To monitor liver health and systemic inflammation; to ensure clinical decisions are well informed and multidimensional	Cross-referenced with poor sleep, ↑ CRP, and ↑ resting heart rate from wearable data

HbA1c—glycated hemoglobin; LDL—low-density lipoprotein; HDL—high-density lipoprotein; CRP—C-reactive protein; ALT—alanine transaminase; AST—aspartate transaminase; GGT—gamma-glutamyl transferase; HRV—heart rate variability; BPM—beats per minutes; ↑—elevated.

**Table 4:** Integrated nutritional monitoring parameters and clinical triggers in the NutriMonitCare System.

Monitored Domain	Parameter	Data Source	Personalized Clinical Insight	Trigger for Intervention/Clinical Action
Anthropometrics	Weight, BMI	Withings Body+ Smart Scale	Tracks weight loss progress and risk of malnutrition	Plateau or excessive loss → evaluate energy intake; BMI < 18.5 → assess malnutrition risk
	Fat mass/muscle mass	Withings Body+ Smart Scale	Differentiates quality of weight loss; muscle loss may reflect protein deficit	↓ Muscle Mass + stable fat % → increase protein intake or physical activity
	%TWL/%EWL	Calculated from weight data	Quantifies surgical effectiveness	Plateau < 5% monthly or excessive TWL > 15% → reassess caloric/protein intake
Hydration and protein	Intake targets	Clinician-defined; patient-tracked	Supports tissue repair and metabolic balance post-surgery	Inadequate adherence → adjust daily goals; reinforce hydration and protein-focused diet
Subjective symptoms	Fatigue, satiety, GI symptoms	Patient self-reports	Reflects nutritional tolerance and energy adequacy	Persistent fatigue → assess for iron/protein deficiency or timing of meals
Biochemical markers	Vitamin B12	Laboratory results	Prevents neuropathy and anemia	<200 pg/mL → B12 supplementation; recheck levels within 1–3 months
	Ferritin/Hemoglobin	Laboratory results	Detects iron-deficiency anemia	Ferritin < 30 ng/mL or Hb < 11 g/dL → iron therapy and dietary adjustment
	25(OH) vitamin D	Laboratory results	Monitors bone health and immune function	<20 ng/mL → vitamin D3 supplementation + calcium intake assessment
	Albumin	Laboratory results	General nutritional marker for protein-energy status	<3.5 g/dL → evaluate protein intake, absorption, or inflammation
Decision layer	Automated alert mechanisms	System dashboard (rule-based logic)	Ensures closed-loop monitoring with prompt clinical action	Triggered by abnormal trends (e.g., ↓ protein intake, weight plateau) → Review plan

BMI—body mass index; TWL—total weight loss; EWL—excess weight loss; Hb—hemoglobin; GI—gastrointestinal; ↓—decrease. → indicate suggested clinical actions derived from the associated parameter values or trends.

## Data Availability

The original contributions presented in this study are included in the article and Supplementary Materials. Further inquiries can be directed to the corresponding authors.

## Supplementary Materials

The following supporting information can be downloaded at: https://www.mdpi.com/article/10.3390/nu17152542/s1, Supplementary File S1: TIDieR Checklist for the development and preliminary evaluation of the NutriMonitCare system.

## Abbreviations

The following abbreviations are used in this manuscript:

AI	Artificial intelligence
ALT	Alanine transaminase
AST	Aspartate transaminase
API	Application programming interface
ASMBS	American Society for Metabolic and Bariatric Surgery
BMI	Body mass index
BS	Bariatric surgery
BSCDT	Bariatric surgery clinical decision tool
CRP	C-reactive protein
CGMs	Continuous glucose monitors
EASO	European Association for the Study of Obesity
EHR	Electronic health record
EMA	Ecological momentary assessment
ERAS	Enhanced recovery after surgery
EWL	Excess weight loss
GAD-7	General anxiety disorder-7
GDPR	General data protection regulation
GI	Gastrointestinal
GGT	Gamma-glutamyl transferase
Hb	Hemoglobin
HbA1c	Glycated hemoglobin
HDL	High-density lipoprotein
HL7 FHIR	Health level 7 fast healthcare interoperability resources
HR	Heart rate
HRV	Heart rate variability
ICD-10	International classification of diseases
IoT	Internet of Things
LDL	Low-density lipoprotein
NICE	National Institute for Health and Care Excellence
PHQ-9	Patient health questionnaire-9
RYGB	Roux-en-Y gastric bypass
SG	Sleeve gastrectomy
SOAP	Subjective objective assessment plan
SpO_2_	Peripheral oxygen saturation
SRQR	Standards for Reporting Qualitative Research
TIDieR	Template for Intervention Description and Replication
TWL	Total weight loss
