# Supplementary material for: Integrated Medical and Digital Approaches to Enhance Post-Bariatric Surgery Care: A Prototype-Based Evaluation of the NutriMonitCare System in a Controlled Setting
Source: Nutrients. 2025 Aug 2;17(15):2542. doi: 10.3390/nu17152542 (PMC12348797; doi:10.3390/nu17152542)
Supplement: Supplementary file 1 [file nutrients-17-02542-s001.zip › nutrients-3769641-supplementary.pdf]

Supplementary File S1

TIDieR Checklist for the development and preliminary evaluation of the NutriMonitCare system

This checklist has been completed to support the reporting of the NutriMonitCare system as described in the as described in the present article.

Item No	Checklist Item	Description in this study
1	Brief Name	The intervention is named NutriMonitCare system, as introduced in Section 2.
2	Why (Rationale, theory, or goal)	Section 2.1 explains the clinical and methodological rationale for using a digital tool to support personalized post-bariatric care.
3	What (Materials)	Described in Sections 2.2 and 2.3. Includes Withings devices, clinical dashboards, patient apps.
4	What (Procedures)	Monitoring protocols, data flows, and feedback loops are described in Section 2.3.
5	Who provided	Clinical providers involved include endocrinologists, bariatric specialists, and nutritionists, as described in 2.1 and 2.2.
6	How	Digital monitoring via wearable devices and dashboard interfaces detailed in Section 2.3.
7	Where	Controlled laboratory environment with simulated use cases. Clarified in Section 2.5.
8	When and how much	Simulated monitoring sessions follow weekly/monthly routines; discussed in Section 2.2.2.
10	Tailoring	Interventions are dynamically adjusted based on monitored patient trends. See Sections 2.2.3 and 2.3.
11	Modifications	System allows real-time updates and parameter personalization. Described in Section 2.3.3.
12	How well (Planned)	System includes alert mechanisms and compliance tracking modules. See Section 2.3.3.
	How well (Actual)	Due to the prototype phase, the system is evaluated under simulated conditions. See Section 2.5.
